# Genetic Continuity and Change Among the Indigenous Peoples of California

**DOI:** 10.1038/s41586-023-06771-5

**Published:** 2023-11-22

**Authors:** Nathan Nakatsuka, Brian Holguin, Jakob Sedig, Paul E. Langenwalter, John Carpenter, Brendan J. Culleton, Cristina García-Moreno, Thomas K. Harper, Debra Martin, Júpiter Martínez-Ramírez, Antonio Porcayo-Michelini, Vera Tiesler, M. Elisa Villapando-Canchola, Alejandro Valdes Herrera, Kim Callan, Elizabeth Curtis, Aisling Kearns, Lora Iliev, Ann Marie Lawson, Matthew Mah, Swapan Mallick, Adam Micco, Megan Michel, J. Noah Workman, Jonas Oppenheimer, Lijun Qiu, Fatma Zalzala, Nadin Rohland, Jose Luis Punzo Diaz, John R Johnson, David Reich

**Affiliations:** 1 Department of Genetics, Harvard Medical School, Boston, Massachusetts 02115, USA; 2 Harvard-MIT Division of Health Sciences and Technology, Boston, MA 02115, USA; 3 Department of Anthropology, University of California at Santa Barbara, Santa Barbara, CA 93106, USA; 4 Department of Human Evolutionary Biology, Harvard University, Cambridge, MA 02138, USA; 5 Department of Anthropology, Biola University, La Mirada, CA 90639, USA; 6 Instituto Nacional de Antropología e Historia, Sonora, Hermosillo, México; 7 Department of Anthropology, The Pennsylvania State University, University Park, PA 16802, USA; 8 Department of Anthropology, University of Nevada, Las Vegas, NV 89154, USA; 9 Instituto Nacional de Antropología e Historia, Mexicali, Baja California, México; 10 Universidad Autónoma de Yucatán, Facultad de Ciencias Antropológicas, Mérida, México; 11 Instituto Nacional de Antropología e Historía, Morelia, Michoacán, México; 12 Howard Hughes Medical Institute, Harvard Medical School, Boston, MA 02446, USA; 13 Santa Barbara Museum of Natural History, Santa Barbara, CA, 93105, USA; 14 Broad Institute of Harvard and MIT, Cambridge, MA, 02142, USA

## Abstract

Prior to colonialism, California harbored more language variation than all of Europe, and linguistic and archaeological analysis have led to many hypotheses to explain this diversity ^1^. We report genome-wide data from 79 ancient Californians and 40 ancient northern Mexicans dating to 7400–200 years before present (BP). Our analyses document long-term genetic continuity between people living on California’s Northern Channel Islands and the adjacent Santa Barbara mainland coast from 7400 BP to modern Chumash groups represented by individuals who lived around 200 BP. The distinctive genetic lineages that characterize present-day and ancient people from Northwest Mexico increased in frequency in Southern and Central California by 5200 BP, documenting northward migrations that are candidates for spreading Uto-Aztecan languages prior to the dispersal of maize agriculture from Mexico ^2–4^. Baja Californians share more alleles with the earliest Central Californian in the dataset than with later Central Californians, potentially reflecting an earlier linguistic substrate, whose impact on local ancestry was diluted by later migrations from inland regions ^1,5^. After 1600 BP, ancient Channel Islanders lived in communities with effective sizes similar to those in pre-agricultural Caribbean and Patagonia, and smaller than those on the California mainland and in sampled regions of Mexico.

## Introduction

People have lived in California since at least 13000 years before present (BP, all dates calibrated in what follows) based on archaeological evidence from the Northern Channel Islands off Southern California ^6^ and the mainland, which were occupied by peoples speaking Chumashan languages at the time of European contact ^1^. California also harbors some of the highest linguistic diversity of any region in the Americas, which is relevant to understanding human population relationships as language often correlates with movements of people ^7^. Linguistic diversity among Native Californians includes multiple major groupings, which have varying divergence time estimates, some exceeding 6000 BP. In the north are Algic (e.g. Yurok), Athabascan (e.g. Hupa), and Yukian (e.g. Yuki). In the central coast and interior valley is Utian (e.g. Miwok and Ohlone) ^8^. On the northern Channel Islands and mainland there is Chumashan. In southern California there is Uto-Aztecan (e.g. Tongva/Gabrieliño and Payómkawichum/Luiseño). Lastly, there are smaller language families (e.g. Yuman-Cochimí) and isolates (e.g. Washo) ^1^. How these divergent language families came to be in such close proximity needs to be understood in a larger context as migration in one direction or another must have been responsible for some families, such as Algic and Uto-Aztecan, that have very wide dispersals, in these cases extending far beyond California. The Uto-Aztecan language family in particular is one of the most geographically widespread families in the Americas, ranging from Shoshone in Idaho to Pipil in Costa Rica and covering the central and west coast of Mexico and the American Southwest. There are many hypothesized homelands, including the Great Basin ^9^, California’s central valley ^10^, the Sonora desert ^11^, central Mexico (from which it has been suggested to have spread with maize farming) ^12^, and southern Arizona to Northern Mexico ^13^, with different types of linguistic and archaeological evidence adduced for each model.

**Figure 1.**
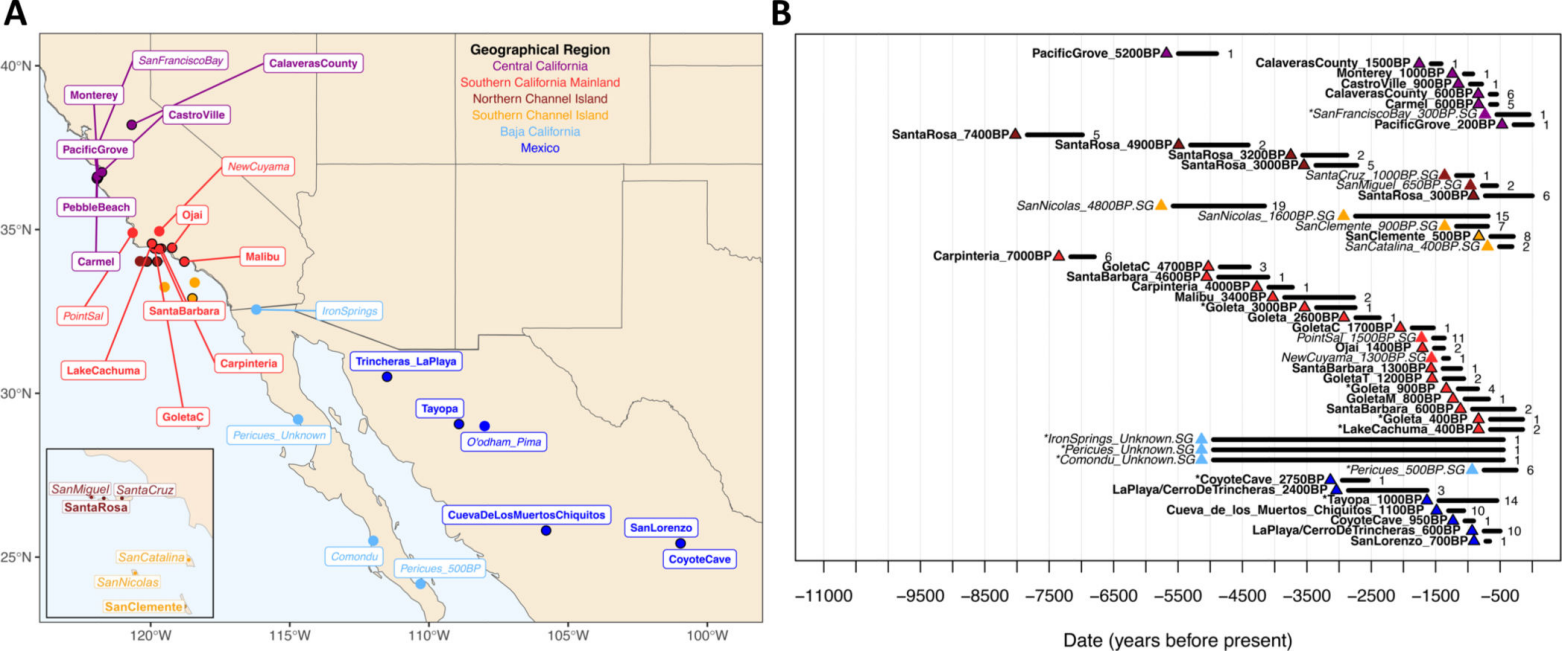
Summary of data. **A)** Locations of analyzed ancient individuals. Newly reported in bold, previously reported in italics. Purple=Central California, red=Southern California mainland, dark red=Northern Channel Islands, orange=Southern Channel Islands and nearby mainland, light blue=Baja California, blue=Mexico excluding Baja California. All data are ancient except O’odham. Map is made with open source data and software using the R packages maps, sf, rnaturalearth, ggplot2, and ggrepel. **B)** Dates of analyzed individuals. Width is the full range of all radiocarbon dates for the group (marine reservoir calibrated, Methods); * indicates no radiocarbon date. Number of individuals per site on the right.

We report data from 119 individuals, including 79 from 7400–200 BP in Central and Southern California, and 40 from 2900–500 BP in Northwest and North Central Mexico (Figure 1; Supplementary Data File 1) (in this paper, we frequently refer to present-day political entities such as US or Mexican states, but caution that modern boundaries artificially divide Indigenous culture areas). To obtain these data, we extracted DNA, generated single and double stranded DNA libraries treated to remove characteristic damage signatures associated with ancient DNA, and enriched for mitochondrial DNA and ~1.2 million single nucleotide polymorphisms (SNPs) across the genome. We sequenced the enriched products on Illumina instruments and evaluated the authenticity of the data, leading us to restrict analyses to 112 newly reported individuals with no evidence of substantial modern contamination (Online Methods and Supplement). We combined these data with previously published ancient and present-day data.

Consistent with previous work ^14^, we find that the earliest DNA sequenced from the Chumash region in California going back to at least around 7400 BP is most closely related to modern South Americans and the ~12800 BP Clovis culture-associated Anzick individual in Montana (“Southern Native American” (SNA) ancestry) ^15^ (Box 1). Genetic clustering of the individuals is correlated with geography; it also correlates with language, with branches enriched in groups likely to have spoken Chumashan, Uto-Aztecan, and Utian. We find evidence for large-scale movement of genetic lineages characteristic of ancient and modern individuals from Northwest Mexico into both South and Central California by at least ~5200 BP. This raises the possibility that this movement was responsible for spreading Uto-Aztecan languages, documenting a period of major migration from the south not associated with farming, and undermining the argument that agriculture need have brought these languages ^12^. Finally, we document a strong genetic relationship of the earliest Central California individual from Pacific Grove ~5200 BP to ancient individuals from Baja California, providing support for the theory that people speaking languages from an earlier linguistic substrate were once dispersed across large parts of California, and that the human populations of the region were later transformed by new migrants who changed both the genetic and linguistic landscapes.

### Ethics and Inclusion Statement

This research was carried out in consultation with Indigenous communities and other stakeholders in California and Mexico, with multiple engagements occurring before sampling, as well as return-of-results meetings prior to paper submission. Co-authors including different subsets of NN, JJ, BH, PL, JLPD, and DR participated in consultations with members of the Chumash, Tongva, Ohlone, and several Mexican communities, with the goal of ensuring that the manuscript reflected community perspectives (additional details in Methods section and Supplementary Data File 1). Our study includes co-authors who not only contributed to the scientific work but are also members of communities with connections to ancient individuals. The final manuscript addresses topics that were emphasized by community members as being of particular interest, including understanding how ancient peoples on the Channel Islands and mainland related to each other, understanding the relationships between ancient peoples in California and those of nearby regions, and understanding the processes that produced the historical distribution of Indigenous languages. We emphasized in these presentations that scientific discovery is a dynamic and iterative process that builds on itself, and that this study is not the final word even on a scientific level, as additional studies will inevitably refine and improve the models and interpretations here. We also emphasized that genetic ancestry is very different from identity which is often based on social relationships rather than biological ties; genetic findings should never be seen as challenging cultural identity.

### Overview of Genetic Data

**Extended Data Figure 1.**
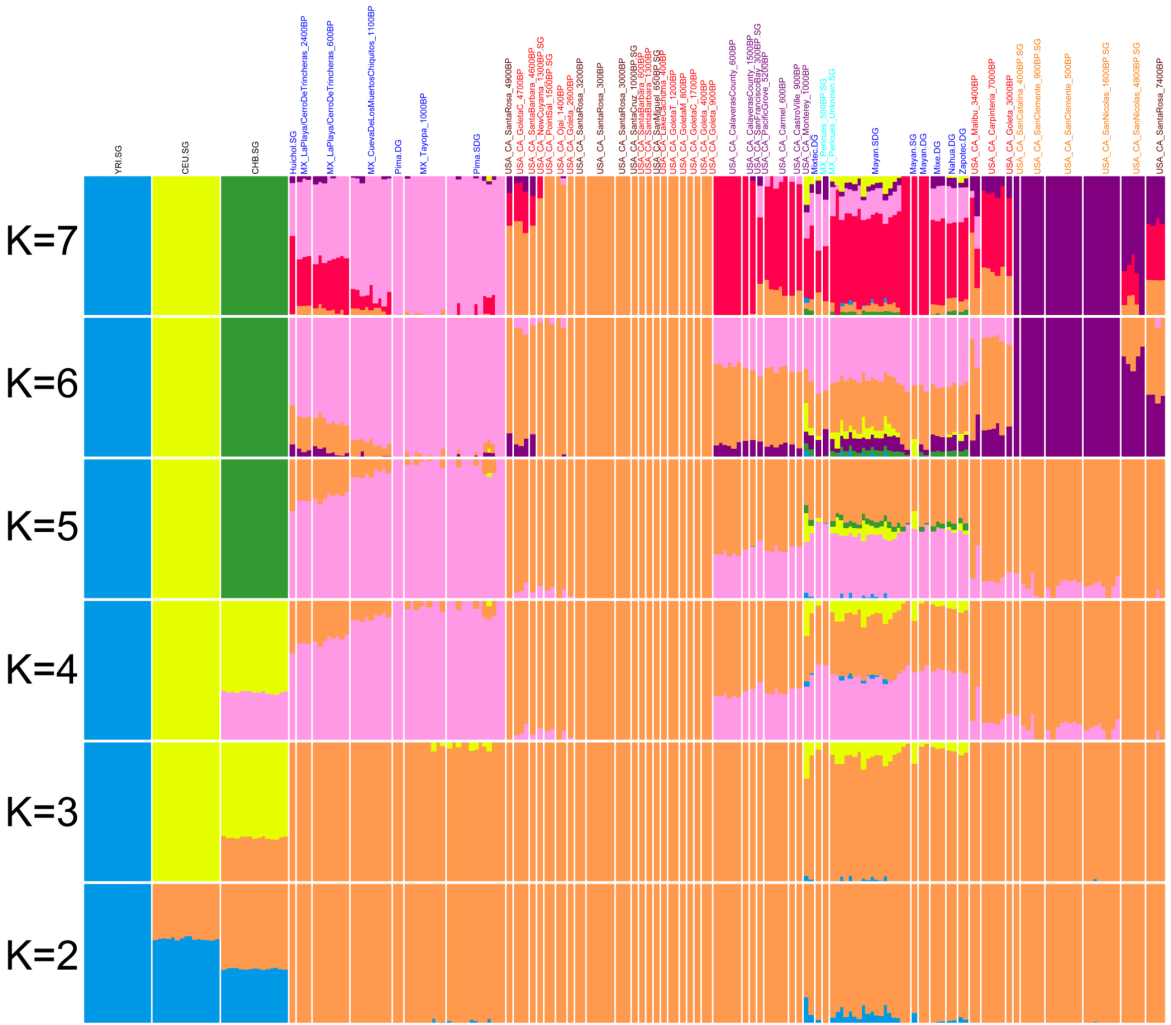
ADMIXTURE plot at different K values. Purple=Central California, red=Southern California mainland, dark red=Northern Channel Islands, orange=Southern Channel Islands and nearby mainland, light blue=Baja California, blue=Mexico excluding Baja California.

**Extended Data Figure 2.**
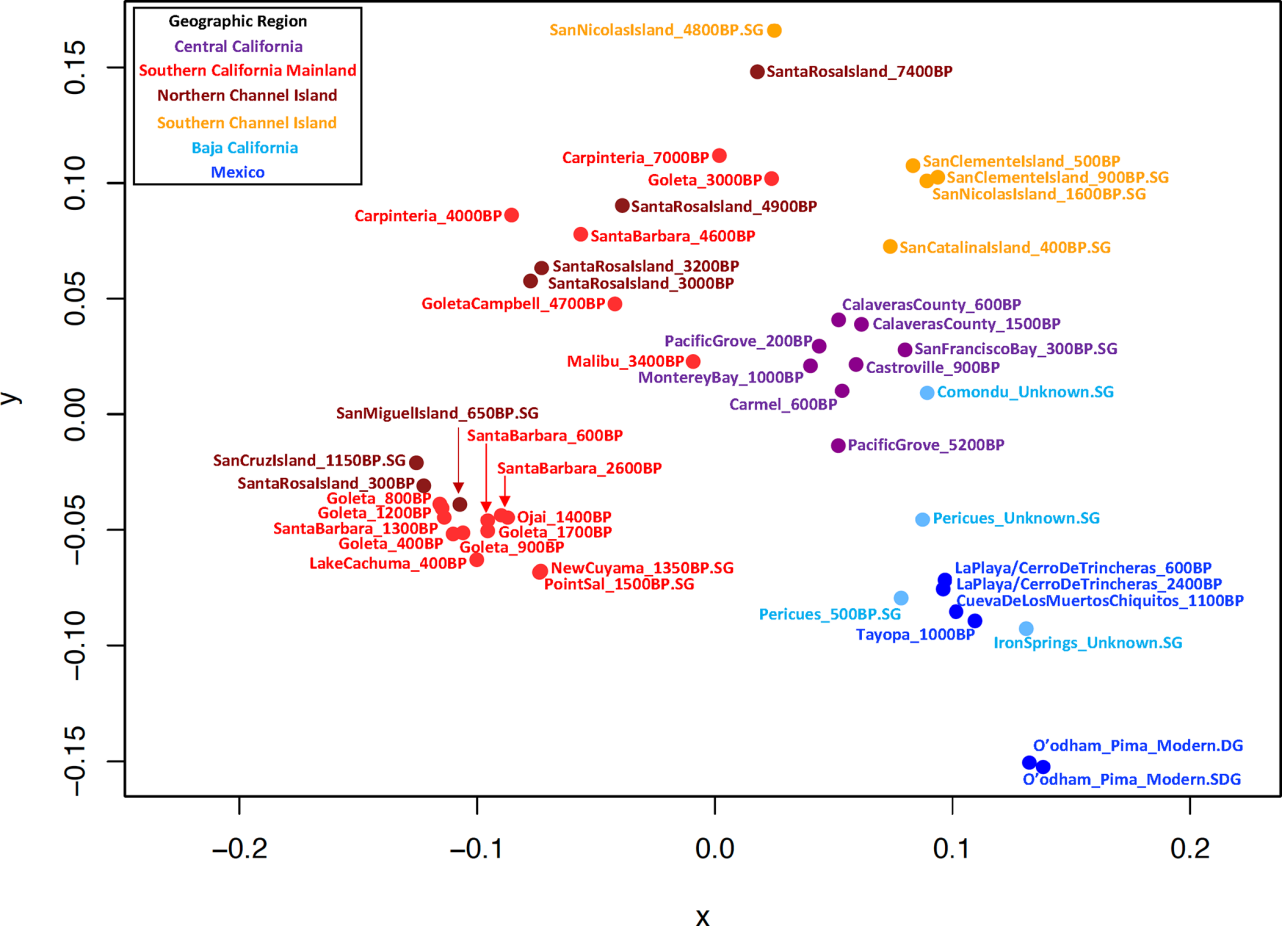
MDS plot of groups created using a matrix of inverted outgroup-*f*_*3*_ statistics (distances = 1-*f*_*3*_*(Mbuti; Group1, Group2)*). Purple=Central California, red=Southern California mainland, dark red=Northern Channel Islands, orange=Southern Channel Islands and nearby mainland, light blue=Baja California, blue=Mexico excluding Baja California.

**Extended Data Figure 3.**
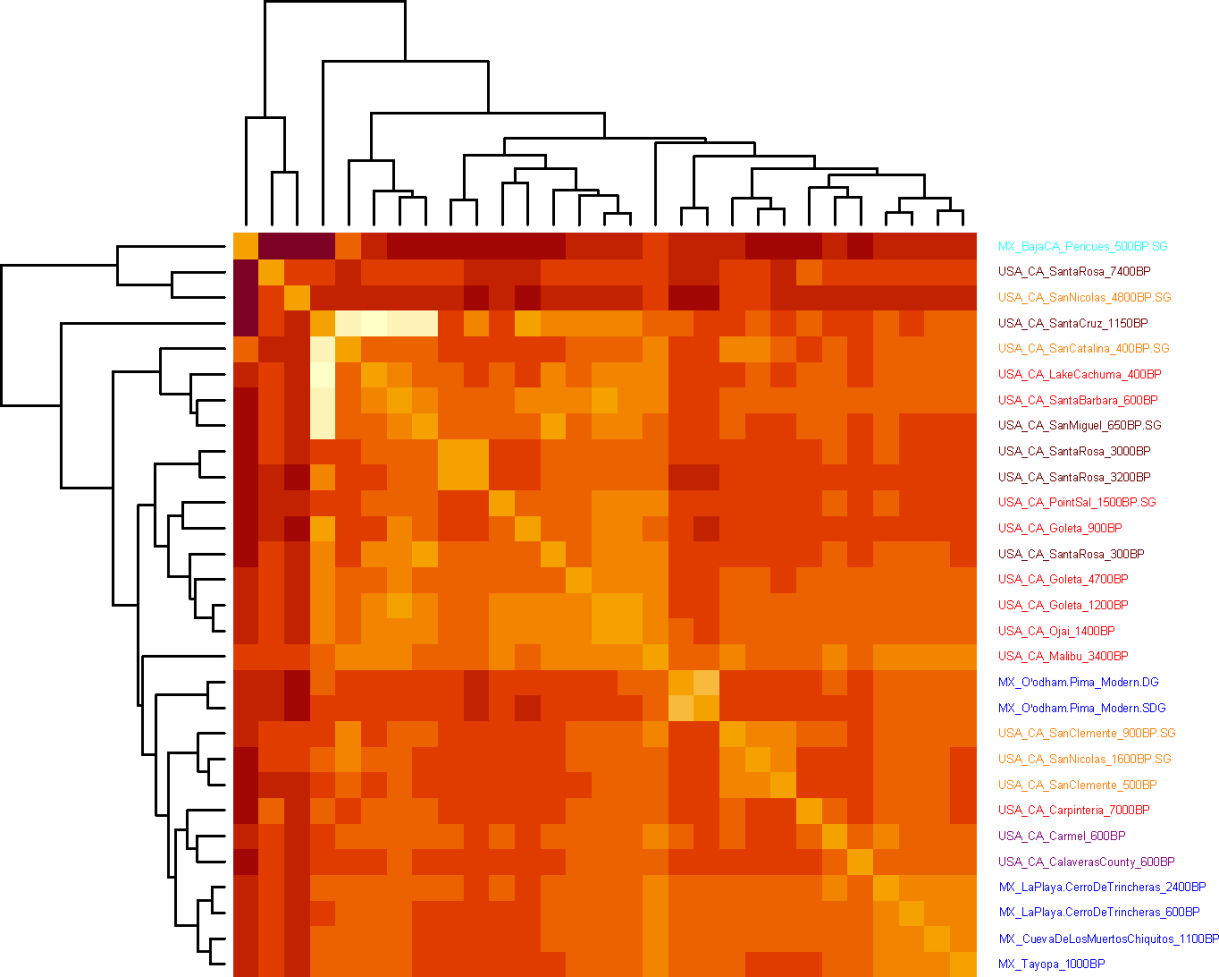
Heatmap of pairwise F_ST_. F_ST_ between groups was estimated using smartpca. Only groups with at least 2 individuals of greater than 100,000 SNP coverage were used. Heatmap and dendrogram were created in R with symm=T. Supplementary Data File 3 shows F_ST_ values. *Purple=Central California, red=Southern California mainland, dark red=Northern Channel Islands, orange=Southern Channel Islands and nearby mainland, light blue=Baja California, blue=Mexico excluding Baja California.*

**Figure 2.**
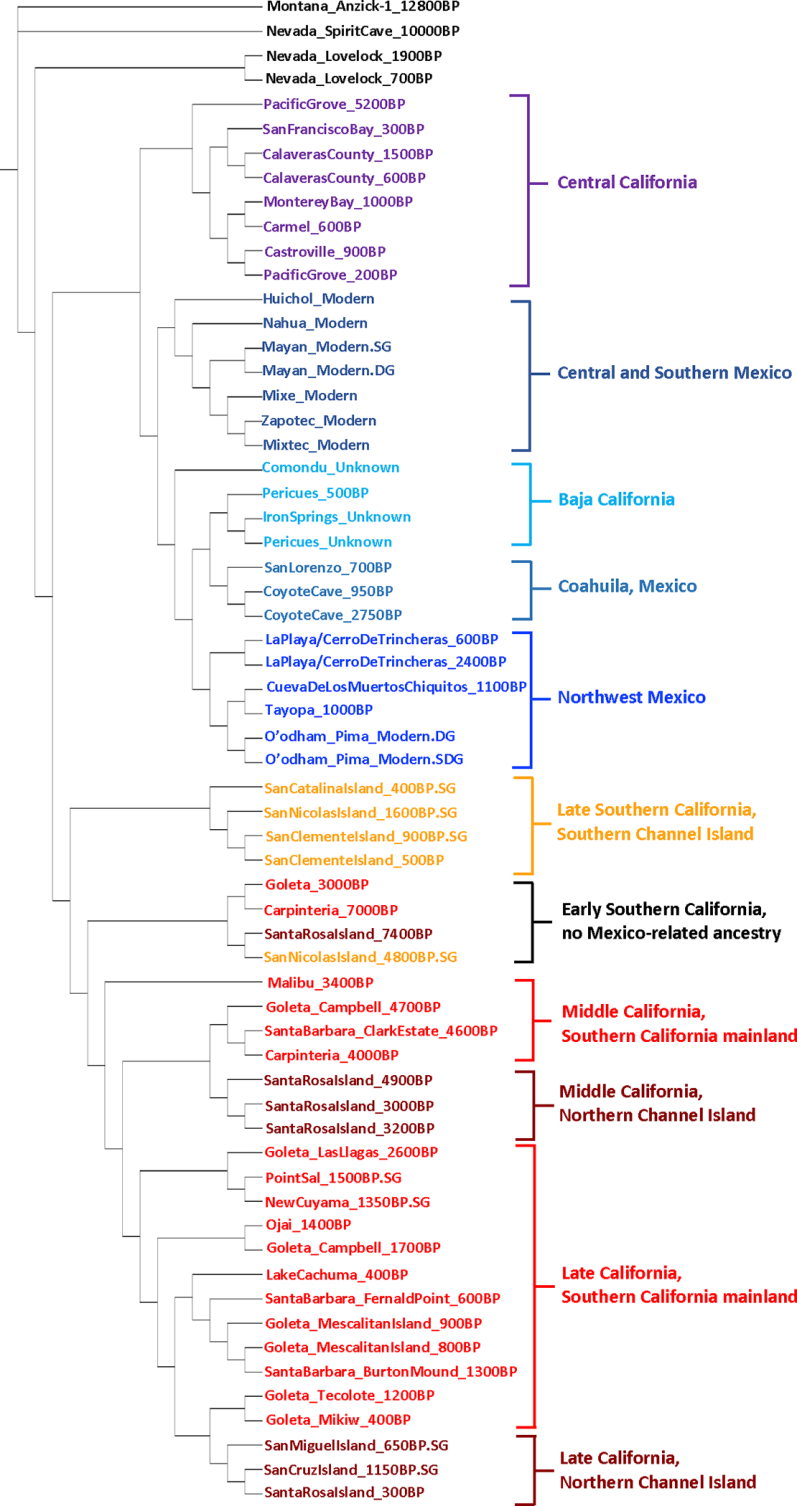
Neighbor-joining tree of groups. Tree created using a matrix of inverted outgroup-f_3_ statistics (distances = 1/f_3_(Mbuti; Pop1, Pop2)) with USA-MT_Anzick-1_12800 BP as the outgroup. Designed using Itol.

We grouped individuals based on their archaeological site and age and found the individuals in these groups to be genetically homogeneous relative to other groups using *qpWave* (Supplementary Data File 2). To cluster groups, we created a neighbor-joining tree (Figure 2), performed unsupervised ADMIXTURE (Extended Data Figure 1), and used Multi-Dimensional Scaling (MDS) (Extended Data Figure 2) based on an “outgroup-*f*_*3*_” matrix of statistics that measures the amount of shared genetic drift between two groups “Pop1” and “Pop2”: *f*_*3*_*(Mbuti; Pop1, Pop2)*. We also computed pairwise F_ST_ (Extended Data Figure 3 and Supplementary Data File 3). The clusters obtained by these procedures correlate to geography and time. From a geographical perspective, we observed differentiation of Northwest Mexico, Baja California, two Southern California regions (roughly Northern Channel Islands which was genetically similar to the Southern California Mainland, and the Southern Channel Islands), and Central California. Geography-based clustering can be an indicator of local population continuity, with later populations descended in substantial part from earlier ones. In some cases, genetic clustering was more correlated with time than with location, a pattern that can be indicative of cross-regional migrations.

Mitochondrial haplogroup frequencies in California show variability over time. Of the individuals over 3500 BP with enough data to make a determination, 30/36 had an A2 haplotype, nearly all from the Santa Barbara Channel Islands and adjacent mainland, with the 6 non-A2 haplotypes all from mainland Southern California (Supplementary Data File 1). After 3500 BP, only 35/91 carry A2 haplotypes, with B2, C1b, C1c, C5b, D1, and D4h3a all represented, consistent with the whole genome evidence of movement of lineages into California from outside. All of the Mexico individuals were younger than 3500 BP and only 5/44 had A2 haplotypes, with B2, C1b, C1c, C5b, and D4h3a also represented.

All Y chromosomes were Q1b1a except for one from the North Mexican site of Cueva de los Muertos Chiquitos. This differs from the much higher rate (~1/3) of Q1a2a in very ancient (>5000 BP) South Americans ^16^ (Supplementary Data File 1).

### Spread of Lineages Characteristic of Mexico into California Prior to the Advent of Agriculture

The time dependency of some of the clustering in the neighbor-joining tree as well as the changes in mitochondrial haplogroups suggest ancestry change over time. To quantify these patterns, we compared the most ancient individuals (~7400 BP) from the northern Channel Island of Santa Rosa (called *wima*’ in the Samala or Ineseño Chumash language) to the more recent ones using statistics of the form *f*_*4*_*(Mbuti, X; Santa Rosa Island 7400BP, Santa Rosa Island <7400BP)*, for Santa Rosa Island groups in each time period, and found that almost all statistics are consistent with 0 (|Z| < 3) for populations X outside of California (Supplementary Data File 4). The only exceptions are a significant genetic affinity of the younger Santa Rosa Island groups to several groups in the south. This includes ~500 BP Pericues individuals from Baja California that plausibly had recent gene exchange with groups related to those in the Channel Islands; it also includes ~1000 BP groups from Sonora in Northwest Mexico (LaPlaya/CerroDeTrincheras and Tayopa); and finally it includes a group in Northern Durango Mexico (Cueva de los Muertos Chiquitos). The *MX_LaPlaya/CerroDeTrincheras_600 BP* individuals gave the most consistent signals of extra affinity to the later Santa Rosa Island individuals, in keeping with them being the most geographically proximal to California. Significant signals were also present when comparing 7000 BP individuals from Carpinteria (on the California mainland coast across the Santa Barbara Channel from Santa Rosa Island) relative to 600 BP individuals from the same area, and comparing the earliest Southern Channel Island individuals (~4800 BP) to the latest ones (~900 BP), with *MX_LaPlaya/CerroDeTrincheras_600 BP* individuals again showing greater affinity to the later groups relative to the earlier ones.

**Figure 3.**
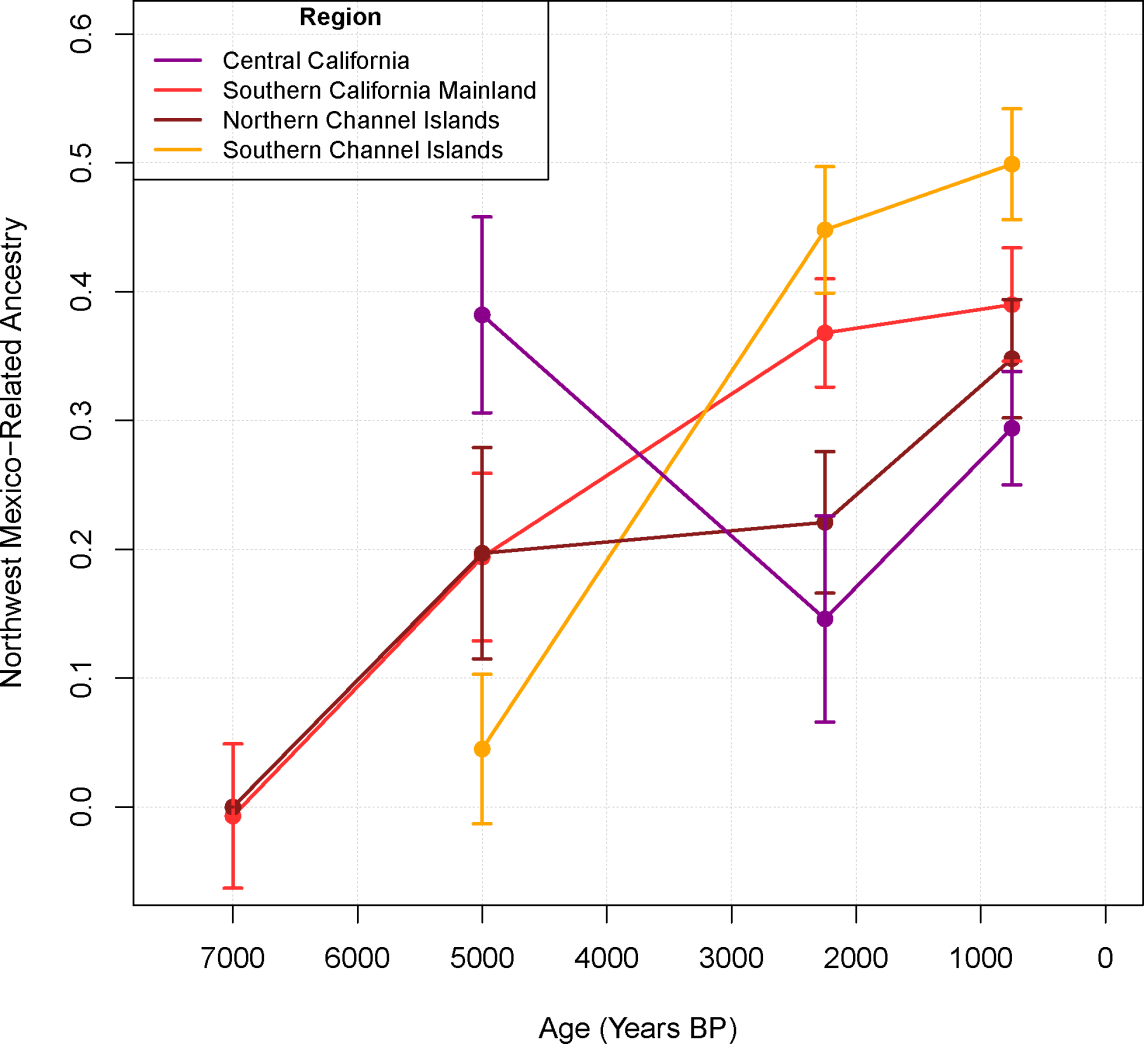
Northwest Mexico-related ancestry at different regions over time, from qpAdm. Each data point represents mean MX_LaPlaya/CerroDeTrincheras_600 BP-related ancestry in a bin of time (8000–6000 BP, 6000–4000 BP, 4000–1500 BP, and <1500 BP) with number of individuals for Central California, Southern California Mainland, Northern Channel Islands, and Southern Channel Islands for each time bin as (0, 6, 5, 0, 1, 5, 2, 19, 1, 6, 7, 15, 15, 30, 5, 17), respectively. Bars represent ±1 standard error, derived from a weighted block jackknife over 5-Mb blocks.

We used *qpAdm* to estimate ancestry in the California individuals as a mixture of sources related to the two proxies highlighted by the preceding analyses: *USA-CA_SantaRosa_7400 BP* and *MX_LaPlaya/CerroDeTrincheras_600 BP* (Figure 3). *qpAdm* is designed to give unbiased estimates even if the relationships are distant. We estimate Mexico-related ancestry of 20±8% in *USA-CA_SantaRosa_4900 BP* (± is one standard error), 22±6% in *USA-CA_SantaRosa_3200 BP*, 23±6% in *USA-CA_SantaRosa_3000 BP*, and 37±5% in *USA-CA_SantaRosa_300 BP*, with all models fitting at p>0.05, (Supplementary Data File 5). In contrast, *USA-CA_Carpinteria_7000 BP*, *USA-CA_SanNicolasIsland_4800 BP*, and *USA-CA_Goleta_3000 BP* are consistent with being directly descended without admixture from earlier groups in the same region (the estimates of Mexico-related ancestry are not significantly different from zero: −1±6%, 5±6%, and −3±11%; p>0.05 for all), consistent with them being a clade and clustering with the oldest Santa Rosa Island individuals, and suggesting the migration is south-to-north (Supplementary Data File 5). (Note: the *USA-CA_Goleta_3000 BP* individual has not been radiocarbon dated and thus could be older than 3000 BP.) When *SanNicolas_4800 BP* or *Carpinteria_7000 BP* were used as sources instead of *SantaRosa_7400 BP*, the estimates were not significantly different (Supplementary Data File 5).

These statistics show that there was gene exchange between people in California and peoples related to those of Northwest Mexico in the second half of the Holocene beginning at least by the 4900 BP date of *USA-CA_SantaRosa_4900 BP*. O’odham speakers (Pima) currently occupy the region in Mexico where the individuals that maximize these affinities reside, and the modern O’odham in our dataset have similar genetics to the ancient NW Mexicans (Figure 2, Extended Data Figure 2, Supplementary Data File 4). The O’odham language belongs to the Uto-Aztecan language family ^10^, and based on the degree of language diversity in the region, the geographic distribution of languages, and knowledge about the rate at which languages evolve, linguists have argued that the great majority of ancient Northwest Mexicans almost certainly spoken Uto-Aztecan languages by 2900–500 BP, the time period of the ancient Mexicans in our dataset ^2–4,17^. Mexico-related ancestry increased over time, reaching the highest levels in the later populations of the Southern Channel Islands (Figure 3). The highest proportion of Mexico-related ancestry occurred in later Southern Channel Island (San Nicolas, San Clemente, and San Catalina) individuals (44–51%), consistent with the observation that Indigenous people in that area at the time of colonial contact spoke an Uto-Aztecan language (Nicoleño). The present-day people in the area, the Tongva (some refer to themselves as Gabrieliños), also speak a closely related Uto-Aztecan language variety. In contrast, Northern Channel Islands Chumash groups speak a language from an unrelated family, and show correspondingly less genetic affinity to northwest Mexican Uto-Aztecan language speakers ^1^.

A remarkable aspect of the spread of ancestry related to Mexican Uto-Aztecans 5200–2000 BP is its geographic extent, with evidence appearing all the way from Central to Southern California and potentially even Baja California (Baja California could not be tested rigorously given the absence of a time transect, but when modeled as mixture of *USA-CA_SantaRosa_7400 BP* and *MX_LaPlaya/CerroDeTrincheras_600 BP*, the Baja California groups all were inferred to have over 60% Mexico-related ancestry). Further evidence that this must have been mediated in large part by migration into California is that we observe no evidence of an increase in California-related ancestry in Mexico. Modeling *MX_LaPlaya/CerroDeTrincheras_600 BP* as a mixture of groups related to *MX_LaPlaya/CerroDeTrincheras_2400 BP* and *USA-CA_Carpinteria_7000 BP*, we obtained a well-fitting model of *LaPlaya/CerroDeTrincheras_600 BP* as descending only from *MX_LaPlaya/CerroDeTrincheras_2400 BP* (−0.5±2.3% *USA-CA_Carpinteria_7000 BP* ancestry; Supplementary Data File 5). Taken together, our results document over 5,000 years of movement of ancestry from people related to modern Northwest Mexican Uto-Aztecan speakers into the Channel Islands and mainland.

A notable exception to this pattern of monotonically increasing affinity to NW Mexico over time is a ~5200 BP individual from central California (*USA-CA_PacificGrove_5200 BP*), which could be well-modeled as having 38±8% Mexico-related ancestry (Supplementary Data File 5), with later Central Californians in the dataset having similar or lower ancestry proportions. Although based on a sample size of one, these results demonstrate unambiguously the presence of Mexico-related ancestry by this time. A non-monotonic pattern is what would be expected from a south-to-north migration by ~5200 BP followed by mixture with other (northern) groups without Mexican relatedness.

These findings show that ancestry related to that common in ancient and present-day people in NW Mexico began spreading at least as far north as Central California beginning at least 5,000 years ago, documenting demographically significant mid-Holocene gene flow between the two regions prior to the spread of agriculture (starting ~4100 BP) ^9,18^. Further evidence for the presence of Mexican-related ancestry in California before the spread of maize-based agriculture northward from Mexico into the US Southwest comes from the presence of the Santa Rosa Island individual ~4900 BP and three Goleta individuals ~4700 BP with significantly elevated (20±8% and 19±7%) Mexico-related ancestry. A ~1900 BP group from Lovelock Cave, Nevada (Great Basin region) ^19^ showed one significant signal in comparisons between more recent California individuals and more ancient ones (Z=3.3 for affinity to *SantaBarbara_900 BP* relative to *SantaBarbara_4600 BP*). Although this could potentially reflect a random statistical fluctuation due to multiple hypothesis testing (Supplementary Data File 4), it is a strong enough signal to provide tentative evidence of population movement. More individuals from Great Basin groups would be necessary to test the extent to which the migration affected the Great Basin as well as California, and also to test if the impacts on California were mediated through the Great Basin.

The strongest case for agriculture being the vector for a northward spread of Uto-Aztecan languages from central Mexico to the western United States has been the argument that the expansion of farming is the only process that could have been demographically transformative enough to propel language change ^20^. In this argument, agriculture led to an increase in population size in the US Southwest, which propelled individuals north into California even though agriculture never spread there. However, our analysis finds that major migration from south to north affected Central California by ~5200 BP, a time before agriculture began spreading and coinciding with the period when linguists have argued that “Old Uto-Aztecan” had reached the San Joaquin Valley before being displaced by Yokutsan languages ^1,10^– suggesting that the pre-5200 BP migrations could have been the events that brought Uto-Aztecan languages to the region. Importantly, however, our data also document a later increase of Mexico-related ancestry in the Southern California Mainland and Southern Channel Islands between 5000 to 3000 BP, which is notable as the California individuals in our dataset are primarily in the region occupied by speakers of the Takic sub-group of the Northern Uto-Aztecan branch, while the Northwest Mexico individuals are primarily in the region occupied by speakers of the Piman sub-group of the Southern Uto-Aztecan branch. The date of the split of these two branches is estimated to be older than 7000 BP by some reconstructions ^9^, and 5000–4000 BP in other reconstructions ^11,13^; our findings of south-to-north migrations into California both before 5200 BP and from 5000 to 3000 BP could be consistent with both reconstructed split times.

How should our genetic findings inform the debate about the likely homeland of Uto-Aztecan languages, beyond undermining the strongest argument in favor of the theory of an origin among central Mexican agriculturalists by showing that the spread of agriculture was not the only demographically significant south-to-north migration? One group of linguistic reconstructions have suggested that Proto-Uto-Aztecan languages were spoken by hunter-gatherers living between southern Arizona and northern Mexico (immediately to the northeast of the *MX_LaPlaya/CerroDeTrincheras* individuals), in a woodland/grassland homeland in proximity to montane forests, based on evidence that the reconstructed proto-language contained words for animals and plants from this region (e.g. agave, long-needled pine, hawk, and owl) ^13^. Our genetic finding of south-to-north migrations into California beginning before 5200 BP and continuing until at least 3000 BP—alongside archaeological evidence of material culture exchange between Mexico and California (e.g. the spread of the contracting stem dart point ^21^ and turquoise ^22^) at least 4000 years ago ^23^—increases the weight of evidence for this theory. Conversely, the fact that we do not observe a significant increase in Great Basin (Lovelock Cave-related) or California Central Valley-related ancestry (Supplementary Data File 5) in northern Mexico argues against either a Great Basin ^9^, or a California Central Valley origin ^10^.

### Genetic Continuity and Immigration in Ancient Central California

**Extended Data Figure 4.**
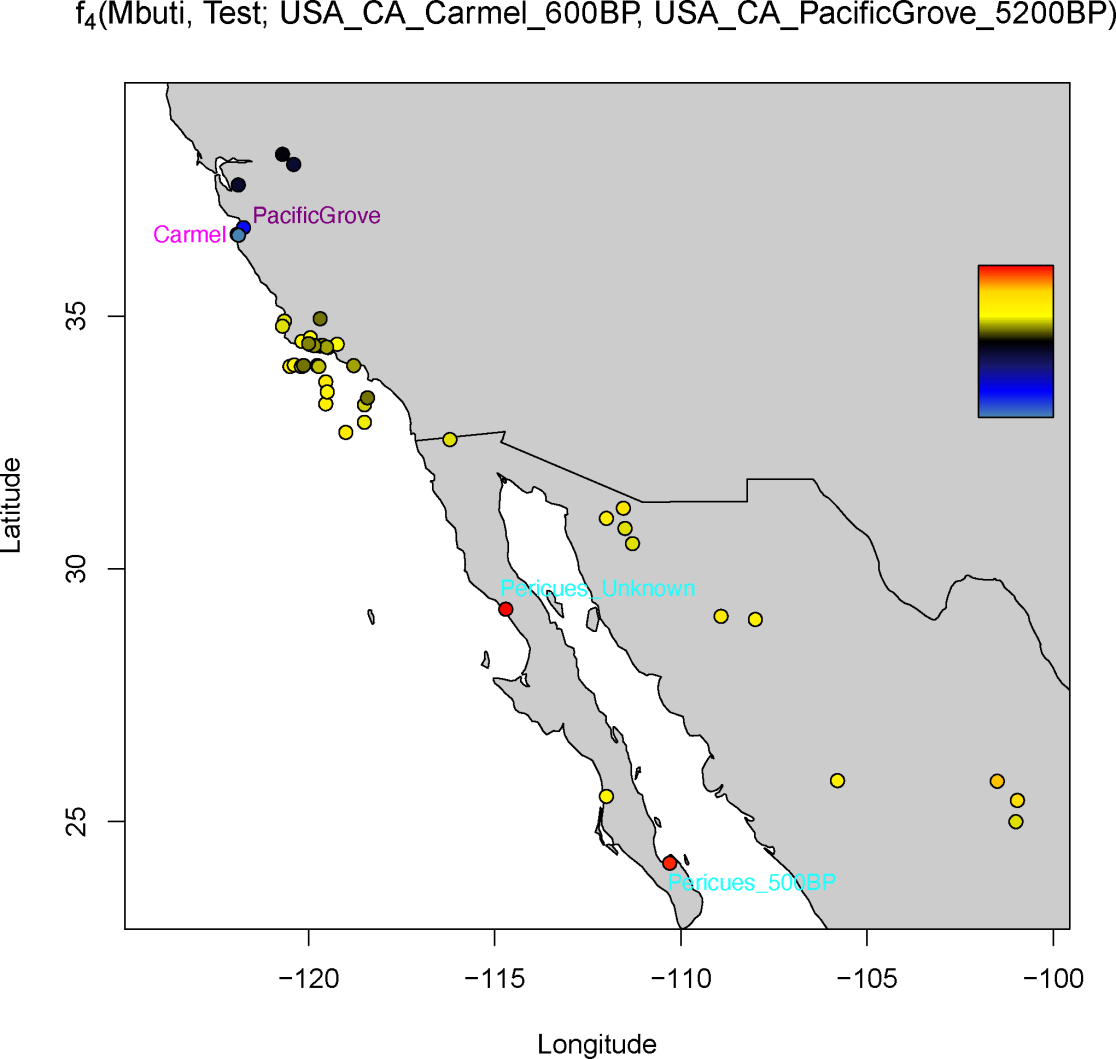
Map of statistics of the form *f*_*4*_*(Mbuti, Test; USA-CA_Carmel_600BP, USA-CA_PacificGrove_5200BP)*. Dots in red show greater genetic affinity to *PacificGrove_5200BP* relative to *Carmel_600BP*, while dots in black and blue have greater affinity to *Carmel_600BP*. Points were jittered to allow better visualization. Figure is generated with open source data and software in R with ggplot2 and the ‘fields’ and ‘RcolorBrewer’ libraries.

We assessed the ancestry of the oldest Central California individual by comparing her to the oldest Santa Rosa Island individuals with the statistic *f*_*4*_*(Mbuti, Test; USA_CA_PacificGrove_5200 BP, USA_CA_SantaRosa_7400 BP)*. The most significant attraction to *PacificGrove_5200 BP* is with *MX_CA_Pericues_500 BP.SG* (Z=6.46, f_4_=0.00413), a relatively isolated group that lived at the southern tip of Baja California in present-day Mexico. This signal is as strong when comparing *PacificGrove_5200 BP* to *USA_CA_CalaverasCounty_1500 BP* (Z=6.47, f_4_=0.00384, for attraction to *Pericues_500 BP*), a group ~180 miles to the east of Pacific Grove and likely within the territory occupied seasonally by speakers of Washo ^1,24,25^. This signal has no evidence of being driven by NW Mexico-related ancestry in Pericues, because it is of similar proportions in the later Central California individuals (*PacificGrove_200 BP*, *MontereyBay_1000 BP*, *Carmel_600 BP*, and *Castroville_900 BP* have between 24 to 42% NW Mexico-related ancestry), and *PacificGrove_5200 BP* still has a significant attraction to *Pericues_500 BP* (2.1<Z<4.3, 0.00209<f_4_<0.00427) relative to these groups (Extended Data Figure 4 and Supplementary Data File 4). Technical differences between shotgun vs. capture ancient DNA methods are also not likely to be artifactually causing this effect, because the groups being compared for their affinity to the shotgun-sequenced Pericues groups both had 1240K capture processing and thus should not have a differential affinity to shotgun sequenced groups (as would be expected if one group had shotgun processing and the other had capture). This raises the possibility that the Pericues-related ancestry decreased over time, with the caveat that the ~5200 BP data point is only one individual and more individuals are needed to assess the true distribution of the ancestries over time.

The Baja California-related signal in the 5200 BP Central California individual is potentially consistent with a previous hypothesis of an earlier linguistic substrate widespread in California and Baja California (Figure 1A) that was broadly replaced later in time in Central California by speakers of Utian languages coming from inland to the coast approximately 4000 BP ^1,5,26,27^. This would plausibly have been accompanied by migrations into the region within this time period, as anthropological evidence shows that language changes are often mediated by movements of people ^28^. One possible source for this migration is Calaveras county in the Eastern Central Valley, as we observe a genetic affinity of *CalaverasCounty_600 BP* for *Carmel_600 BP* relative to *PacificGrove_5200 BP* (Z=3.7, f_4_=0.0016), indicating potential migration between these regions from 5200 BP and 600 BP (*Carmel_600BP* was used for comparison, because it was geographically the closest to Pacific Grove while also having multiple high-coverage individuals sequenced). However, the individuals from Calaveras County were thought to also have spoken a non-Utian language (Washo) ^24,25^, so migration from this region does not neatly fit into the Utian migration hypothesis. Denser sampling from 5000–3000 BP would be necessary to determine with more confidence the geographic origin of the source population that moved into the Central California coast and provide a clearer picture of the history of this region.

Movement of people to Central California did not fully displace the original ancestry in the region as later Central Californians have ancestry related to the *PacificGrove_5200 BP* individual, consistent with previous evidence of a degree of local continuity ^29^ (*f*_*4*_-statistics show significant affinity of *PacificGrove_5200BP* to younger Central California coast individuals relative to *SantaRosa_7400BP*, though the statistics are non-significant or only marginally significant when compared with Calaveras County groups (2.1<Z<3.0), possibly due to lack of power (Supplementary Data File 4)). When we modeled later Central California individuals as a mixture of *PacificGrove_5200 BP* and *CalaverasCounty_600 BP*, we found well-fitting models with between 55±14% to 76±9% *PacificGrove_5200 BP*-related ancestry (Supplementary Data File 5), showing that the largest fraction of ancestry is consistent with having deep local roots, similar to the pattern in Southern California.

### Relationship of Ancient Californians to the Earliest Sequenced Native Americans

Early Holocene individuals from Brazil, Chile, and Nevada (*Brazil_LapaDoSanto_9600 BP*, *Chile_LosRieles_12000 BP*, and *USA_NV_SpiritCave_10000 BP*) share more alleles with the Clovis culture-associated *USA-MT_Anzick_12800 BP* individual than with later populations in the same regions ^16,19^. Our analysis shows that this specific affinity to an individual from the Clovis culture persisted for many more millennia in the Chumash region of California than it did in any other sampled regions of the Americas. Symmetry *f*_*4*_-statistics and outgroup-*f*_*3*_ statistics assessing the rate of allele sharing with Anzick relative to an outgroup such as *USA-AK_USR1_11500 BP* show that the earliest California individuals (*USA_CA_SantaRosa_7400 BP* and *USA_CA_Carpinteria_7000 BP*) have affinity similar to those of the earliest individuals from Brazil, Chile, and Nevada, and significantly more affinity to Anzick relative to *Peru_Lauricocha_8600 BP* (Z>3.3) and *Peru_Cuncaicha_9000 BP* (Z>2.4) (Supplementary Data File 4). This suggests that the ancient California individuals descend from an early spread of people with affinity to *USA-MT_Anzick_12800 BP*, and bear more affinity to this lineage than the earliest individuals of similar age from the Central Andes ^14,16^.

**Extended Data Figure 5.**
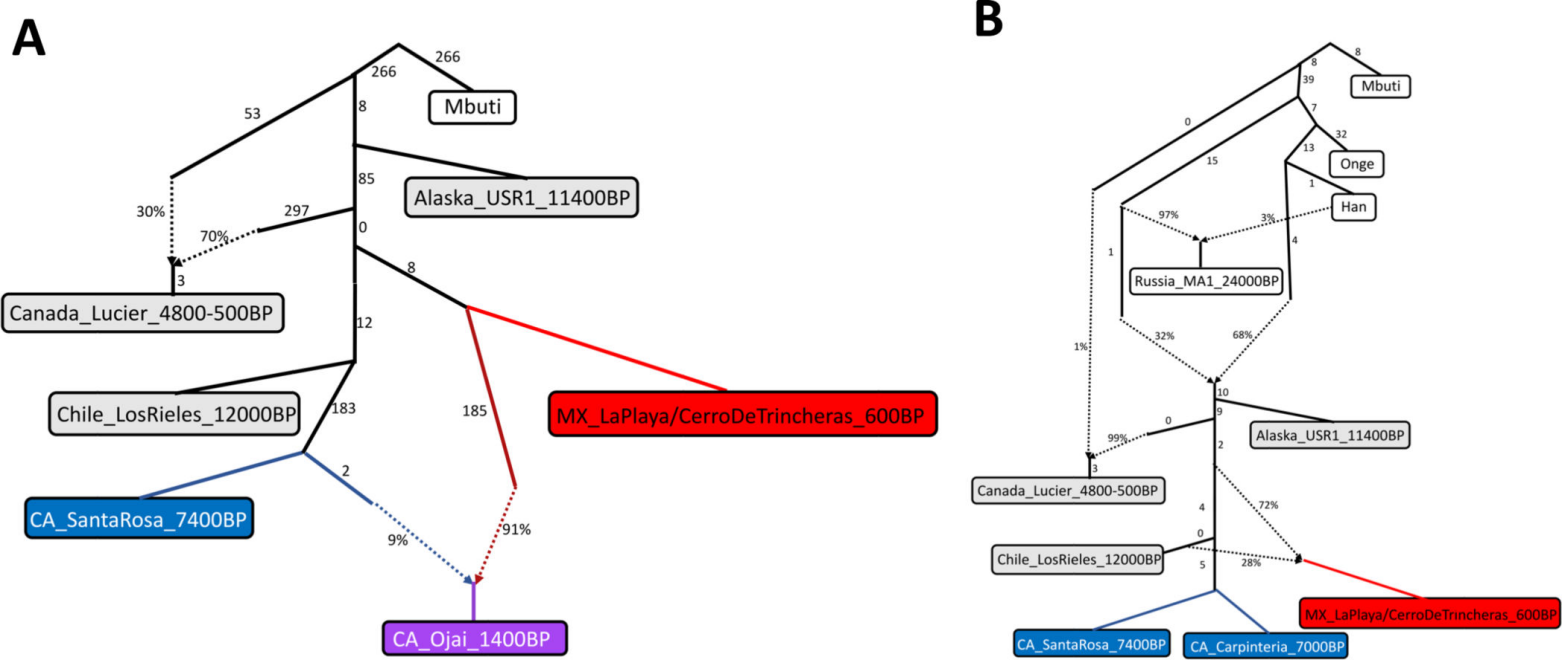
Admixture graphs. **A)** Example admixture graph testing for attraction to *Canada_Lucier_4800-500 BP*. This graph fits with a maximum |Z-score| of 2.81. We tested all subsequent graphs replacing CA_Ojai_1400BP with another ancient California group (Supplementary Data File 4). **B)** Admixture graph consistent with relationships between ancient California and Mexico groups. This graph fits with a maximum |Z-score| of 2.98. All graphs we explore require a lineage more basal than that of *Chile_LosRieles_12000 BP* to fit the Mexico individuals, although we caution that the total space of admixture graph topologies is too large to explore exhaustively so we are making no claim that these particular graphs are correct (only that they are plausible and not ruled out by the data). The basal ancestry into *Canada_Lucier_4800-500 BP* is present to account for known European contamination.

We assessed whether the ancient California and Mexico individuals in our dataset, particularly the younger ones, show any evidence of ancestry from the other main branch of Northern Native American (NNA) ancestry, related to ancient Southern Ontario individuals *Canada_Lucier_4800–500 BP*. We computed a statistic sensitive to this, *f*_*4*_*(Mbuti.DG, Canada_Lucier_4800-500 BP; Test, Chile_LosRieles_12000 BP)*, and found it was consistent with 0 for all ancient California and Mexico groups, providing no evidence for NNA ancestry (Supplementary Data File 4). We also created an admixture graph using *qpGraph* with *Canada_Lucier_4800-500 BP* as an outgroup. We found admixture graphs with plausible fits (all Z-scores<3.0) with almost all ancient California groups not requiring additional ancestry from branches related to *Canada_Lucier_4800-500 BP* (Extended Data Figure 5 and Supplementary Data File 4). The exceptions were *USA_CA_SanClemente_500 BP* and *USA_CA_SanCatalina_400 BP.SG*, which had poor fits (3.05<Z<3.25), though not due to an attraction of them with *Canada_Lucier*. We view these as likely artifacts given the *SanClemente_900 BP* group had a good fit and the *SanCatalina* group could be a poor fit due to technical biases that differ between shotgun sequencing and capture data. Overall, we find no consistent evidence of NNA ancestry in the ancient California and Mexico individuals, in contrast to a previous study ^14^ that modeled intermediate proportions in all the California individuals they reported, all of which we reanalyzed here (Supplementary Data File 4).

### Ancient Indigenous Mexicans Harbored Ancestry from Non-Clovis-Associated Southern Expansions

When we modeled ancient Northwest Mexico individuals, in all fitting admixture graphs (Z<3.0 for the worst residual), the predominant ancestry of the ancient Mexico individuals is more basal (early splitting) than *Chile_LosRieles_12000 BP* and the ancient California individuals, though still less basal than NNA (the best graph we found is presented in Extended Data Figure 5B). This is due to the Mexico groups being on an SNA lineage that is basal to Anzick and does not have the same affinity to Anzick that the Los Rieles and California individuals have. This is also shown by *f*_*4*_-statistics, which show a significant affinity of *USA-MT_Anzick_12800 BP* for *USA_CA_SantaRosa_7400 BP* and *USA_CA_Carpinteria_7000 BP* relative to *MX_Tayopa_1000 BP* (Z=5.6), *MX_Cueva de los Muertos Chiquitos_1100BP* (Z=4.4) and *MX_LaPlaya/CerroDeTrincheras_600 BP*) (Z=5.0) (Supplementary Data File 4). Relative to ancient Mexicans, there is also a significant affinity based on *f*_*4*_-statistics between *USA-MT_Anzick_12800 BP* and *Brazil_LapaDoSanto_9600 BP* (Z=4.8), *Chile_LosRieles_12000 BP* (Z=3.8), and *USA-NV_SpiritCave_10000 BP* (Z=4.1), but they are consistent with 0 in comparisons with *Peru_Cuncaicha_9000BP* (Z=1.1) and *Peru_Lauricocha_8600 BP* (Z=0.2) (the results were also qualitatively the same when using only transversion SNPs; Supplementary Data File 4), suggesting that the earliest Californians might have shared ancestry with the Anzick-related individuals found in Chile, Brazil, and Nevada ^16,19^, while the ancient Mexicans in our dataset might have shared ancestry with the earliest Peruvians. These findings appear superficially similar to those from a study ^30^ that found a contribution from a lineage basal to Anzick in Aridoamerican and some Mesoamerican Mexicans (all of our ancient Mexican individuals were from Aridoamerica). However, the divergent SNA lineage we infer for the Mexico groups is different from the previous findings of “UPopA1” or “UPopA2” linages contributing to Mexicans ^19,30,31^, because both UPopA populations were inferred to be lineages more basal to that of both SNA and NNA, whereas our deep Mexican lineage is consistent with being SNA.

### Relationship of Ancient California to People of Other World Regions

We tested for evidence of Polynesian ancestry based on suggestions that the *tomol* (plank canoe) of the Chumash and Tongva might have had influence from Polynesia ^32^. We used f_4_-statistics to test for genetic affinity between Polynesian (a Hawaiian, an ancient Tongan, or another ancient sample of Polynesian ancestry) and California individuals from 7100 BP to 300 BP relative to *SantaRosa_7400 BP*, and also used *qpAdm* to test for Polynesian ancestry in the California individuals. We found no evidence for Polynesian ancestry in any of the individuals (Supplementary Data Files 4 and 5), consistent with arguments against Polynesian contribution to *tomol* development ^33^. We also tested for excess relatedness to Australasians (“population Y”) using *f*_*4*_*(Mbuti, Onge or Papuan; Test, Mixe)* and found no evidence for it in any of the ancient individuals from California and NW Mexico (Supplementary Data File 4).

We tested the claim of an ancient migration into the Central Andes after ~4200 BP by people distinctively related to ancient Southern California individuals ^16^. We confirm the previously reported signal, and also make a new observation, namely that the signal can only be perceived when outgroups with Mexican-related ancestry are used in *qpAdm*. Thus, when *USA-CA_SantaRosa_7400 BP*, *USA-CA_Carpinteria_7000 BP*, or *USA-CA_SanNicolas_4800 BP* are used as outgroups with no evidence of Mexican ancestry, there is no signal of extra migration into Peru after 4200 BP (p>0.05; Supplementary Data File 6). However, when California groups with Northwest Mexico-related ancestry are used as outgroups, the signal is present (p<0.005). In the publications reporting this finding ^16,34^, California groups with Mexico-related ancestry were used as outgroups. This indicates that the signal, also found by an independent analysis that used Mixe in southern Mexico among the outgroups ^19^, might have been due to a migration of Mexico-related ancestry simultaneously into both the Central Andes and the California Channel Islands after ~4200 BP, or even to Central Andes-related south-to-north migration affecting Mexico after ~4200 BP without new migration into the Central Andes ^35^.

### Community Sizes in the Channel Islands were smaller than in mainland California or Mexico

**Extended Data Figure 6.**
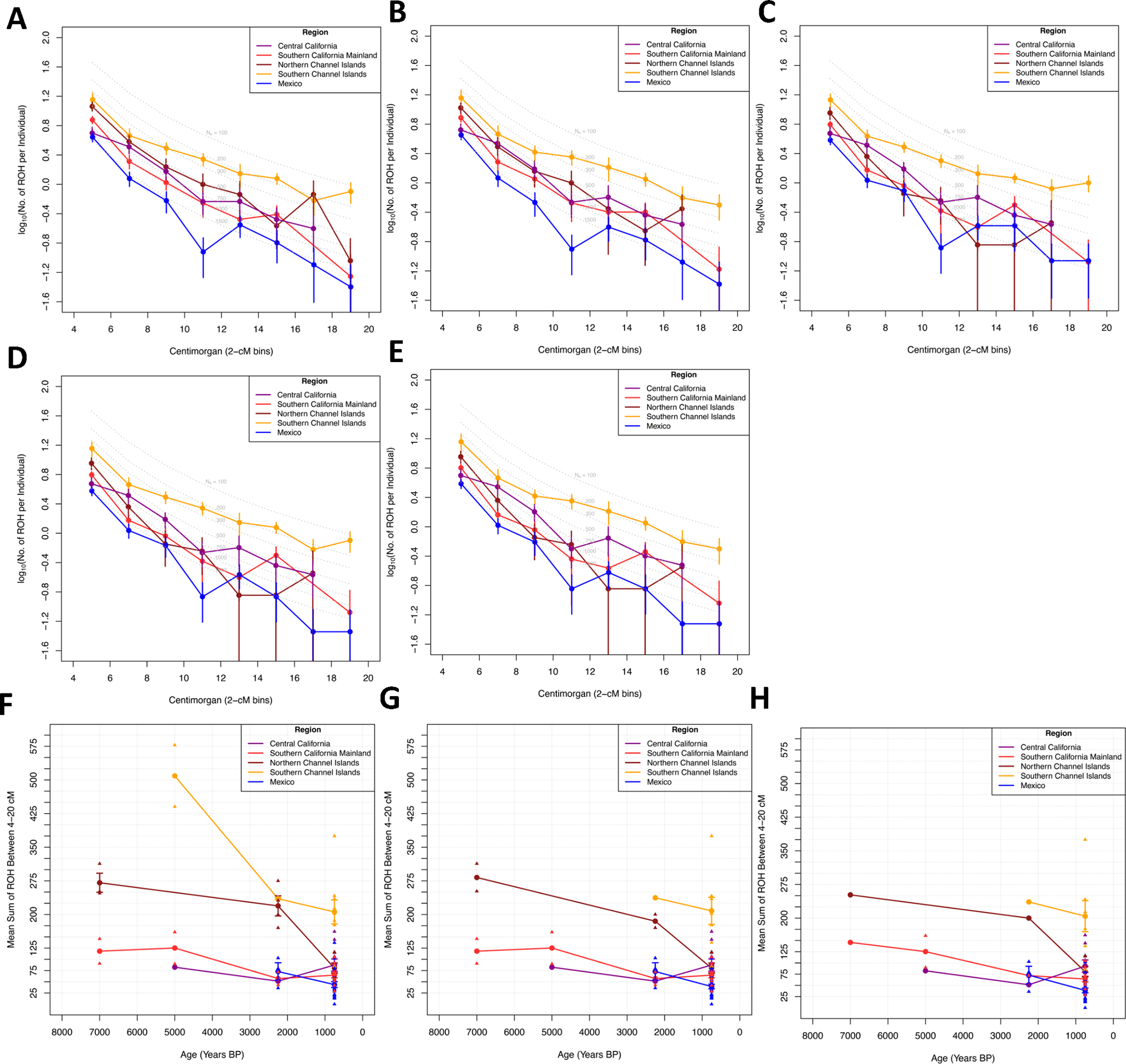
ROH in California and Mexico. **A)** Average rate of ROH segments in different length bins after filtering out individuals with summed 20 cM over 100cM and **B)** after filtering out individuals with summed 20 cM over 50cM. Points with no ROH fragments present in those bins were filtered out. **C)** Average rate of ROH segments in different length bins after filtering out individuals over 1600 BP and **D)** after filtering out individuals with summed 20 cM over 100cM or **E)** over 50cM. **F)** ROH over time where each data point represents the average sum of ROH between 4–20 cM of individuals in a bin of its corresponding time-period (8000–6000 BP, 6000–4000 BP, 4000–1500 BP, and <1500 BP). **G)** ROH over time after filtering out individuals with summed 20 cM over 100cM or **H)** over 50 cM. For F-H, the number of individuals for Central California, Southern California Mainland, Northern Channel Islands, Southern Channel Islands, and Mexico for each time bin are (0,2,3,0,0,1,2,0,2,0,1,2,4,3,3,10,12,7,9,23), (0,2,2,0,0,1,2,0,0,0,1,2,2,2,3,10,12,7,8,22), and (0,1,1,0,0,1,2,0,0,0,1,1,1,1,3,9,11,7,7,21), respectively. For all figures, data are presented as mean values ±1 standard error (no standard errors are presented for points with fewer than 3 individuals).

**Extended Data Figure 7.**
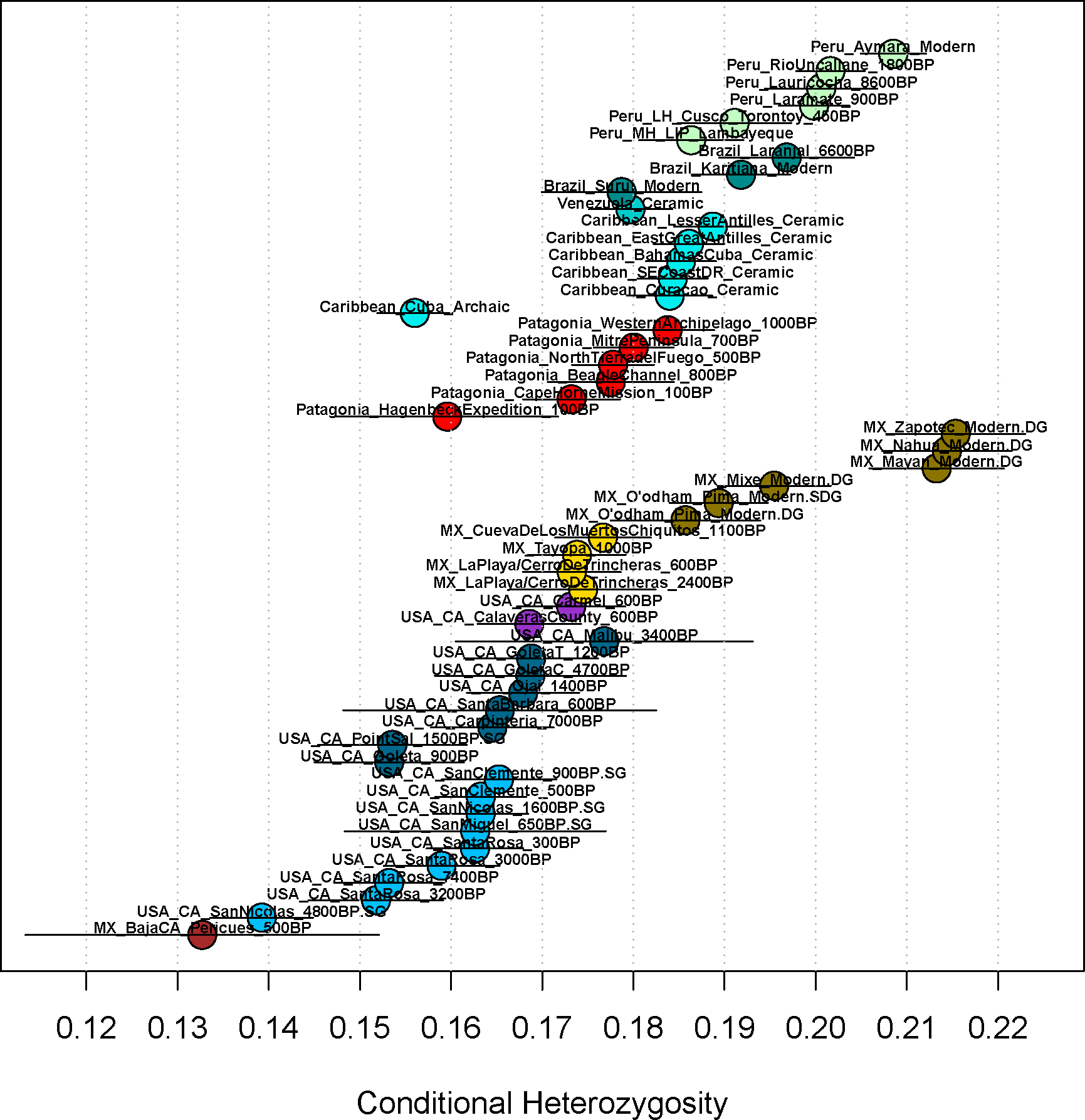
Conditional heterozygosity of groups. Ancient Californian, Mexican, Peruvian, Brazilian, Caribbean, and Patagonian groups and present-day Mexican, Brazilian and Peruvian groups are shown. Only groups with at least 2 individuals could be included in these analyses.

**Figure 4.**
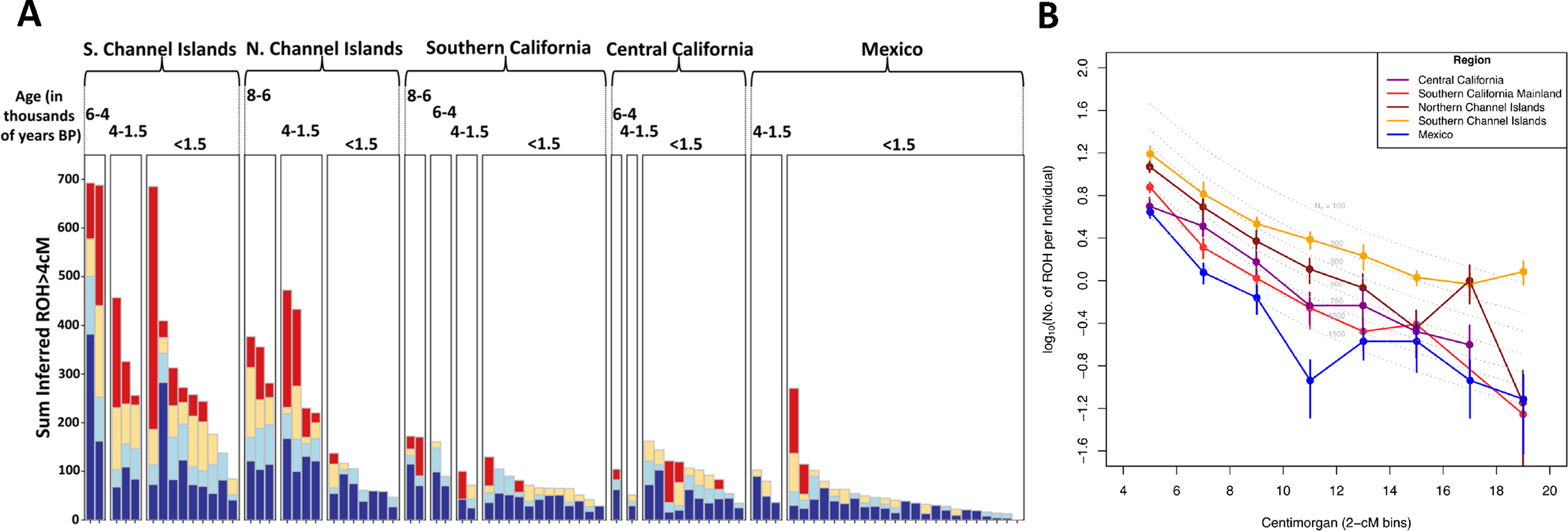
Runs of Homozygosity (ROH) in all ancient Californians and Mexicans. **A)** ROH in all individuals with sufficient coverage. Dark blue indicates sum ROH of 4–8cM fragments; light blue 8–12 cM; tan 12–20 cM; and red 20–300 cM. Different regions grouped by age. Numbers above the bars are the age of the individuals in thousands of years BP. **B)** Average rate of ROH in different length bins for all ancient individuals. Points with no ROH fragments present in those bins are filtered out. For Figure 4B, no filtering was performed; results after filtering out consanguineous individuals or individuals >1600BP are in Extended Data Figure 6.

We analyzed runs of homozygosity (ROH) to estimate effective community sizes of the ancient California and Mexico groups, referring to the size of the mate pool in the last handful of generations. For this purpose we used the software *hapROH*, analyzing 85 ancient individuals with data at over 300,000 SNPs. The most striking patterns are evident in small (4–8 cM) and mid-size (8–20 cM) ROH, which occur at higher rates in the Channel Islands than the mainland of southern and central California (Figure 4A, Extended Data Figure 6 and Supplementary Data File 7), indicating that mothers and fathers of individuals often tended to descend from the same ancestors in the last handful of generations. We estimated effective community size (*N*_*e*_) using the length distribution of ROH at all spatial scales 4–20 cM, which arise from shared ancestry at different time depths in the last 50 generations and make it possible to detect signals of size change and migratory rates with neighboring communities over this temporal scale. When analyzing individuals younger than 1600 BP, and after filtering out individuals with evidence of recent close kin unions (those with ROH fragments larger than 20cM that total more than 50cM), Northern and Southern Channel islands had an estimated *Ne* of 388±42 and 175±13, respectively, similar to pre-agriculture Archaic Caribbean sites ^36^ (232±8) and ancient groups from Patagonia (171±7), Guam (333±11), and Saipan (375±16) (Table 1). Southern California mainland and Central California had *Ne* of 519±49 and 418±39, respectively (Figure 4B and Supplementary Data File 7). In contrast, ancient NW Mexico had *Ne* of 839±74, in line with estimates using modern genomes (Figure 3A of García-Ortiz et al., 2021 ^30^) and similar to estimates for Ceramic-associated Caribbean sites with agriculture ^36^ (681±21) and similar-aged Peruvian groups (817±51). Effective community sizes in the more southern Mexican groups were larger than the more northern ones (CuevaDeLosMuertosChiquitos = 1105±181, Tayopa = 895±142, LaPlaya/CerroDeTrincheras = 605±94, in order from south to north). This could reflect southern settlements having more access to water and fertile land for agriculture, allowing larger communities to develop. Alternatively, these patterns could reflect more frequent exchange of mates between southern villages than between northern villages, without implying that villages in the two regions were different in size from each other. The findings are consistent with analyses of conditional heterozygosity—rates of variation at sites polymorphic in an outgroup (Yoruba from Africa)—as we found lower heterozygosity in the ancient California islands than in any other group, as expected if ancestral variation was lost due to persistently small community sizes (Extended Data Figure 7). However, the sizes of the mate pools in the Channel Islands and Southern California increased over time, as indicated by decreasing ROH (Extended Data Figure 6 and Supplementary Data File 7).

## Conclusion:

The history of Indigenous people in California reflects Late Pleistocene migrations into the region, followed by mid-Holocene south-to-north migrations of people related to Uto-Aztecan speaking groups of Northwest Mexico, and additional migration that affected the Central California coast correlated with ancestry found in inland Central California Valley populations. Our data and analyses demonstrate that the earliest sequenced people in the Chumash region were related to the genetic profile of the Anzick individual of Late Pleistocene age. In-place genetic continuity can be documented through the millennia down to modern Chumash as represented by sequences from 200 BP. There has been substantial debate about whether early speakers of Uto-Aztecan languages originated as hunter-gatherers from the Southwestern US-Northwestern Mexican border area, as maize farmers in central Mexico, or from the Great Basin region of the present-day USA and spread southward ^9,10^. Our results show that ancestry related to present-day Mexican Uto-Aztecan speakers was present in admixed form in Central California at least 5200 BP and in Southern California at least 4900 BP, and provides no evidence for a spread of Central California or Great Basin-related ancestry southward into Mexico. This fits best with the scenario of hunter-gatherers moving both Northwest into California and south into Mexico. Our results provide an alternative vector for the spread of Mexican ancestry to California than the spread of maize agriculture, which was the previous best argument for a south-to-north movement of Uto-Aztecan being associated with agriculture, because the earliest evidence for maize expansion into the Southwest is only ~4100 BP ^9,37^. The finding of this ancestry in Central California at 5200 BP is also consistent with linguistic theories that Uto-Aztecan languages were already spoken in the Central Valley by the Middle Holocene ^1,10^.

It is possible that currently unsampled ancient groups from outside of Southern California and Northwest Mexico (e.g. the western Great Basin ^10^) mixed into both of these regions, which could have produced some of the genetic signals. For example, there is evidence for drought conditions between ~6300 to 4800 BP ^38^, particularly on the coasts and the northwest Mexico desert, while the Great Basin was somewhat more wet in this period. Some linguists have proposed that these conditions led to the diversification of Proto-Uto-Aztecan into its north and south branches, with the northern branch migrating up to the Great Basin region during this time ^38,39^. There is also archaeological evidence for cultural exchange between the Great Basin and southern and central California between 5900 and 4700 BP based on the distribution of *Olivella* grooved rectangle beads produced on the southern Channel Islands and the adjacent southern California coast ^40^ as well as in central California ^41^ and the spread of obsidian throughout these areas ^42^.

We were unable to determine the geographic origin of the migration into Central California after 5200 BP. Collection of genetic data from present-day California Indigenous groups, and co-analysis with ancient data in California and beyond, would provide additional insights. It is important to carry out such research in a way that is engaged with present-day Indigenous descendant groups, following approaches like those taken in this and previous studies, and informed by recent discussions and recommendations concerning ethical analysis of DNA from ancient Indigenous individuals ^14,16,19,29,36^.

## Online Methods

### Ethical approval:

We acknowledge the Indigenous peoples of California and Mexico who supported this study as well as the ancient individuals whose skeletal remains we analyzed. Studies of DNA from ancient individuals can have deep and important implications for present-day groups, because it can reveal information about their ancestors, including their history and interactions with others, and also because the physical handling of the skeletal materials can be sensitive to descendant communities. We performed this study in strong engagement and with participation from local Indigenous communities with closest ties to the ancient individuals we studied. We also performed this study according to ethical guidelines for working with human remains, treating the Indigenous ancient individuals with the respect due to deceased people.

For the California ancient individuals, the ancient skeletal remains we analyzed were curated primarily at the UC Santa Barbara museum. The newly sequenced San Clemente individuals were curated at the Peabody Museum of Archaeology and Ethnology. All ancient California skeletal remains were repatriated to the tribes residing in the region where the ancient individuals originally lived, and the skeletal remains were reburied by the tribes (additional details provided in Supplementary Data File 1). The exception to this were the newly sequenced San Clemente individuals, whose skeletal material were deemed culturally unidentifiable and for which an official federal register notice was posted, with discussions currently ongoing to determine the best approach to repatriation of these ancient individuals. Co-authors JJ, NN, BH, PL, and DR participated in multiple engagements with several Chumash groups in Southern California (author BH is a tribal descendant of the Santa Ynez Band and meetings occurred with permission granted from the Santa Ynez, Barbareño, and Barbareño-Ventureño bands), as well as with the Tongva in Southern California and Ohlone and Esselen groups in Central California. Manuel Armenta, an elder of Santa Ynez band of Chumash Indians and NAGPRA representative for the tribe, gave permission for DNA sequencing. Armenta and his colleague Rex Saint-Onge, met with JJ to formulate research goals. Several of the ancient individuals in Central California were sequenced and studied as part of long-term engagements by the late Gary Breschini, who obtained support for DNA testing by Ohlone tribal members and the late Ella Rodriguez, who was designated by the State Native American Commission as Most Likely Descendant (MLD) for the Monterey Bay area.

In Mexico, all legal authorizations were obtained for this work, sanctioned by the Consejo de Arqueología from the Instituto Nacional de Antropología e History. The research followed their guidance, and was directed by Mexican archaeologists (authors JC, CGM, JMR, APM, EVC, and JLPD). Individuals from Trincheras, La Playa were part of the PIPANOM (Proyecto de investigación de poblaciones antiguas en el norte y occidente de México) Project and curated at different centers of the Instituto Nacional de Antropología e Historia in West and North Mexico. Individuals from San Lorenzo, Tayopa, and Coyote Cave were approved for research by collections committees at the Peabody Museum of Anthropology and Ethnology and the American Museum of Natural History. Individuals from Cueva de los Chiquitos were curated at the Anthropology Department of University of Nevada, Las Vegas. In Mexico, consultation occurred through the Mexican government cultural agencies by authors JLP and JS, including with groups in Northwest Mexico with closer connections to the Indigenous cultures. Information for repatriation of the ancient Mexican individuals held at American institutions to their homelands was provided to the INAH such that repatriation efforts for these individuals are being guided by their cultural agencies.

During all community engagements, results were shared and support was obtained for the data to be made public, with possible implications of this also discussed. With the help of Indigenous community members, we prepared a Frequently Asked Questions (FAQ) document (Supplementary Data File 8) to assist the general public with understanding the findings in this study. This project involved components in both the United States and in Mexico providing Indigenous tribal members and local community members training in genetics, archaeology, and ancient DNA as well as career advice and mentoring. Indigenous community members provided feedback on the manuscript before final publication, with the goal of ensuring sensitivity of the final manuscript to community perspectives.

We emphasized in these presentations that scientific discovery is a dynamic and iterative process that builds on itself, and that this study is not the final word even on a scientific level, as additional studies will inevitably refine and improve the models and interpretations here. We also emphasized that genetic ancestry is very different from identity which is often based on social relationships rather than biological ties; genetic findings should never be seen as challenging cultural identity.

### Direct AMS 14C bone dates:

We generated 54 new direct Accelerator Mass Spectrometry (AMS) ^14^C dates for 54 ancient individuals, which we added to previously reported ^14^C dates for other individuals as well as archaeological context information to provide information on chronology (Supplementary Data File 1).

### Calibration of radiocarbon dates:

All calibrated ^14^C ages were calculated using OxCal version 4.4 using different mixtures of the northern hemisphere terrestrial (IntCal20) ^43^ and marine (Marine20) ^44^ calibration curves. Marine dietary contribution was estimated using stable carbon and nitrogen isotope measurements from collagen (Supplementary Data File 1). Nitrogen is sensitive to the relative importance of marine dietary resources, with δ^15^N values of ~11.5‰ expected for a wholly terrestrial diet and ~22.0‰ expected for a predominately (~90%) marine diet. We used nine categories of calibration curve mixing defined by 10% increments (10–90%), each with an applied uncertainty value of ±10%. For individuals from the Santa Barbara Basin, we used a variable marine ΔR model based on the variable reservoir ages for this region from paired organic and planktonic marine foraminiferal carbonate in laminated varves and linear regression ^68^. For individuals from the Monterey Bay area, we used the nearest published ΔR values from Ingram and Southon 1996 ^69^ (based on modern mollusks). In both cases, ΔR values were recalculated according to the Marine20 calibration curve.

### Ancient DNA laboratory work:

We extracted DNA using a method that is optimized to retain small DNA fragments ^45–47^. We converted the DNA into a form that could be sequenced using a double-stranded library preparation protocol, usually pre-treating with the enzyme Uracil-DNA Glycosylase (UDG) to reduce the characteristic cytosine-to-thymine errors in ancient DNA ^48^. For some libraries, we substituted the MinElute columns used for cleaning up reactions with magnetic beads, and the MinElute column-based PCR cleanup at the end of library preparation with SPRI beads ^49,50^. We enriched the libraries both for sequences overlapping mitochondrial DNA ^51^, and for sequences overlapping about 1.24 million nuclear targets after two rounds of enrichment ^52–54^. We sequenced the enriched products on an Illumina NextSeq500 instrument using v.2 150 cycle kits for 2×76 cycles and 2×7 cycles, or on an Illumina HiSeq X10 instrument using 2×101 cycles and 2×8 cycles, and sequenced up to the point that the expected number of new SNPs covered per 100 additional read pairs sequenced was approximately less than 1.

### Computational processing of initial sequence data:

We merged paired reads that overlapped by at least 15 nucleotides using *SeqPrep* (https://github.com/jstjohn/SeqPrep) taking the highest quality base to represent each nucleotide, and then mapped the sequences to the human genome reference sequence (GRCh37 from the 1000 Genomes project) using the *samse* command of the Burrows-Wheeler Aligner (*BWA*) (version 0.6.1) ^55^. Duplicates were removed with Picard version 2.23.0 (http://broadinstitute.github.io/picard/). We trimmed two nucleotides from the end of each sequence, and then randomly selected a single sequence at each site covered by at least one sequence in each individual to represent their genotype at that position (“pseudo-haploid” genotyping).

### Contamination estimation:

We assessed evidence for ancient DNA authenticity by measuring the rate of damage in the first nucleotide (flagging individuals as potentially contaminated if they had a less than 3% cytosine to thymine substitution rate in the first nucleotide for a UDG-treated library and less than 10% substitution rate for a non-UDG-treated library). We used *contamMix* to determine evidence of contamination based on polymorphism in mitochondrial DNA ^56^, and ANGSD to determine evidence of contamination based on polymorphism on the X chromosome in males ^57^. We also used *ContamLD* ^58^ to assess the rate of contamination in autosomal DNA. We removed (but still report) 7 individuals from analyses with point estimates of more than 7% contamination from *ContamLD*, 5% from ANGSD or 10% from *contamMix* applied to mitochondrial DNA.

### Kinship analyses:

We analyzed all pairs of individuals to determine if any of them had evidence of close genetic relatedness. In these analyses we examined all non-CpG autosomal sites and calculated an average mismatch rate at all SNPs covered by at least one sequence read for both individuals. We then compared these rates to the rate of difference between the two chromosomes in each individual, assumed for this analysis to come from individuals not closely related to each other^59^. We remove from group analyses all individuals inferred to have a first cousin or closer relationship with another individual in the dataset (retaining the higher coverage individual) but analyze them in individual-level analyses.

### Analyses of uniparental haplogroups:

We determined the mtDNA haplogroups for all individuals by analyzing the .bam files, restricting to reads with MAPQ≥30 and base quality≥20. W created consensus sequences with *samtools* and *bcftools* version 1.31 using majority rule and then using *HaploGrep2* with Phylotree version 17. We determined Y chromosome haplogroups with the same filtering as for mtDNA reads. We called haplogroups based on the most derived mutation using the nomenclature of the International Society of Genetic Genealogy (ISOGG) (http://www.isogg.org) version 14.76 (April 2019), and using the methodology reported in Lazaridis *et al.*, 2022 ^60^ using YFull YTree v. 8.09 phylogeny (https://github.com/YFullTeam/YTree/blob/master/ytree/tree_8.09.0.json).

### ADMIXTURE clustering analysis:

Using *PLINK2* ^61^, we first removed SNPs in high linkage disequilibrium using the command –indep-pairwise 50 5 0.5. We removed individuals and genetic variants with high missingness and variants with low minor allele frequency using the command –mind 0.9 –geno 0.5 –maf 0.01. We ran ADMIXTURE ^62^ with 10 replicates, reporting the replicate with the highest likelihood and stopping at K=7 due to the significantly higher cross-validation errors that occur after this point (the CV errors from 2 through 9 are, in order: 0.807, 0.822, 0.824, 0.850, 0.870, 0.878, 0.947, 0.962). We thus show results for K=2 to 7 in Extended Data Figure 1.

### Testing of group homogeneity using *qpWave*:

We used the *qpWave* methodology ^54^ in the ADMIXTOOLS package version 6.0 to determine if there was general genetic homogeneity within groups. We tested all pairs of individuals within each group with 3 outgroups chosen to be in close geographic proximity and age to the test group. Pairs of individuals were considered to be consistent with being genetically homogeneous relative to the outgroups if their p-values were greater than 0.01.

### *f*-statistics:

We used the *qp3pop* and *qpDstat* packages in ADMIXTOOLS version 6.0 to compute *f*_*3*_-statistics and *f*_*4*_-statistics (using the f4Mode: YES parameter). In these statistics, standard errors were computed with a weighted block jackknife over 5-Mb blocks. We computed outgroup *f*_*3*_-statistics of the form *f*_*3*_*(Mbuti; Pop1, Pop2)*, which measures the shared genetic drift between population 1 and population 2. We used these statistics to create an MDS plot and neighbor-joining tree by creating a matrix of outgroup-*f*_*3*_ statistics values between all pairs of populations and converting to distances by either taking the inverse of the values for the neighbor-joining tree, or subtracting the values from 1 for the MDS plot. The MDS plot was generated in R and the neighbor-joining tree was generated using PHYLIP version 3.696’s ^63^ neighbor function and setting *USA_MT_Anzick-1_12800 BP* as the outgroup. The tree was displayed using *Itol* ^64^ with all tree lengths set to ignore.

### F_ST_ Analyses:

We used *smartpca* version 5.0 ^65^ to compute F_ST_ values between all groups with at least two individuals. We used fstonly: YES and inbreed: YES with all other settings left at default. We then used this matrix to create a heatmap using hierarchical clustering-based dendrogram in R with symm=T.

### Admixture graph analyses:

We used the *qpGraph* package ^66^ in ADMIXTOOLS to fit models of population splitting and mixture to the allele frequency correlation statistics (*f*-statistics) relating the different groups. We used a basic graph for Native Americans ^67,68^ and then successively added in additional populations in all combinations allowing up to one admixture from the previously fit groups into the graph. We took the graph with the lowest maximum Z score and then repeated the process, adding another population in until all populations of interest were added. Our process for choosing the added populations was to start with the oldest populations and those known to have the most divergent ancestries and then add the younger populations afterwards. We also explored choosing alternate orders of populations to determine if the final graphs was affected by the order of populations added (they were not).

### Quantitative analysis of mixture proportions using *qpAdm* and *qpWave*:

We used the *qpAdm* methodology ^54^ in the ADMIXTOOLS package version 6.0 to estimate the proportions of ancestry of populations deriving from a mixture of reference populations by assessing the relative shared genetic drift with a set of ‘outgroup’ populations. We set the parameters as details: YES, which reports a normally distributed Z-score for the fit (estimated with a block jackknife), and Allsnps: YES to address the relatively low coverage in many of the samples. P-values were obtained by block jackknife resampling and using a likelihood ratio test (two-sided). A model was considered a plausible fit if P>0.01. For *qpWave* analyses, we analyzed all triplets with *Brazil_LapaDoSanto_9600 BP* or *Chile_LosRieles_12000 BP* as pop1, *Peru_Lauricocha_8600 BP* or *Peru_Cuncaicha_9000 BP* as pop2, and Peruvian, Chilean or Bolivian groups after 4200 BP as pop3 as in Posth et al. and Nakatsuka et al. ^16,34^. We used the following outgroups: *USA-CA_SanNicolas_4800BP.SG*, *USA-CA_Carpinteria_7000BP*, *USA-CA_SantaRosa_7400BP*, *USA-NV_SpiritCave_10000BP.SG*, *USA-MT_Anzick_12800BP.SG*, *Russia_MA1_2400BP.SG*, *Papuan.DG*, and *Karelia_HG.SG* to test for the Anzick-1 relatedness in LapaDoSanto and LosRieles and the California Channel Island groups without evidence of Mexico-related ancestry. To study California Channel Island individual with evidence of Mexico-related ancestry, we replaced *Carpinteria_7000BP* and *SanNicolas_4800BP* in these analyses with *USA-CA_SantaBarbara_600BP* and *USA-CA_SantaBarbara_1500BP*. We used the Allsnps: NO parameter to decrease biases for these analyses and left all other settings as default.

### Conditional Heterozygosity Analyses:

We estimated conditional heterozygosity to infer the cumulative effect of bottlenecks in a population’s history over millennia by examining polymorphisms in Yoruba and at these sites, sampling a random allele from two randomly chosen individuals. We performed these analyses at transversion variants on all groups with at least two individuals per site using POPSTATS (https://github.com/pontussk/popstats) with the September 26, 2018 default settings. We computed this on individuals from this study, ancient Peruvians ^16,34^, Brazilian ^16^, Caribbean ^36^, and Patagonian groups ^69^, as well as on present-day Native American human sequencing data ^70,71^. Statistical significance between different groups was assessed by two-sided student’s T-tests.

### Analyses of Runs of Homozygosity:

We used *hapROH* (version 0.1a8; https://pypi.org/project/hapROH/) to identify ROH ^72^.We used the 1000 Genomes Project haplotype panel as the reference panel with 5,008 global haplotypes. We analyzed the ancient and present-day data of individuals with at least 400,000 SNPs covered to identify ROH longer than 4 cM. We also estimated *Ne* using a maximum-likelihood inference framework for ROH size range of 4–20 cM ^36^. We estimated the confidence interval using the curvature of the likelihood (Fisher information matrix). We used the default settings of hapROH for all analyses. The individuals analyzed are shown in Supplementary Data File 1 and include groups from Guam and Saipan ^73^, Patagonia ^69^, Peru and Bolivia ^34^, and the Caribbean and Venezuela ^36^.

### Map Plotting:

Map in Figure 1A was made using the open-source R packages *maps* (version 3.4.1), sf (version 1.14) ^74^, *rnaturalearth* (version 0.3.4) ^75^, *ggplot2* (version 3.4.3) ^76^, and *ggrepel* (version 0.9.3) ^77^. Extended Data Figure 4 was generated in R using *ggplot2* (version 3.4.3) ^76^, *fields* (version 15.2) ^78^, and *RcolorBrewer* (version 1.13).

## Extended Data

## Supplementary Material

Supplementary Data File 1

Supplementary Data File 2

Supplementary Data File 3

Supplementary Data File 4

Supplementary Data File 5

Supplementary Data File 6

Supplementary Data File 7

Supplementary Data File

Supplementary Information Guide

Supplementary Information

## Figures and Tables

**Table 1.** Effective population size estimates from HapROH.

Group	Pop. Size
Patagonia <1500BP	171 ± 7
S. Channel Islands <1600 BP	175 ± 13
Caribbean_Archaic <3200 BP	232 ± 8
Venezuela_Ceramic 2000-3000 BP	274 ± 25
Guam <800 BP	333 ± 11
Saipan <800 BP	375 ± 16
N. Channel Islands <1500 BP	388 ± 42
Central California <1600 BP	418 ± 39
S. California Mainland <1500 BP	519 ± 51
Bolivia <1700 BP	663 ± 62
Caribbean_Ceramic <1700 BP	681 ± 21
Peru <1800 BP	817 ± 51
Mexico <1600 BP	839 ± 74

See Supplementary Data File 7 for additional calculations. Error bars represent ±1 standard error.

## Data Availability

All sequencing data newly generated in this study are available from the European Nucleotide Archive, accession number: PRJEB66319. Genotype data obtained by random sampling of sequences at approximately 1.24 million analyzed positions are available from Harvard Dataverse at accession number: Z2JD58. The data we are publishing in this study are the “DNA libraries” for each of the ancient individuals we analyzed, which are molecular copies of the original molecules extracted from the ancient individuals whose remains in many cases may no longer be available for scientific study. The data we report are thus not only stored after publication in digital form (the sequences we uploaded), but in molecular form for as long as the libraries are maintained in freezers. This means that more sequences may be generated by those who can support generating a higher quality digital readout of the library, with permission to generate such sequences covered by the present publication. These libraries can only be requested for scholarly use and cannot be used for commercial purposes. If the relevant Indigenous communities request them to be repatriated/reburied, they will no longer be available. In addition, we used the following publicly available datasets: Scheib *et al.* 2018 (ENA: PRJEB25445), Nakatsuka *et al.*, 2020 (ENA: PRJEB37446), Posth *et al.*, 2018 (ENA: PRJEB28961), Fernandes *et al.*, 2021 (ENA: PRJEB3555), Nakatsuka *et al.*, 2020 (ENA: PRJEB39010), Lindo *et al.*, 2018 (ENA: PRJNA470966), Mallick *et al.*, 2016 (ENA: PRJEB9586, ERP010710), Raghavan *et al.*, 2014 (NCBI: SRP029640), and Moreno-Mayar *et al.*, 2018 (ENA: PRJEB29074). Genotype data used in analysis are available at: https://reich.hms.harvard.edu/datasets. The hg19 human genome reference sequence was used for all analyses: https://www.ncbi.nlm.nih.gov/datasets/genome/GCF_000001405.25/.

## Box 1. Key archaeological and linguistic inferences

1. There was substantial genetic continuity between people who lived on the Northern Channel Islands and Santa Barbara region from 7400 BP to modern Chumash represented by 200 BP people.
2. People with ancestry related to Uto-Aztecan speakers in Mexico moved into southern and central California on large scales beginning before 5000 years ago.
3. People related to those in the inland valley arrived on the Central California coast after 5000 years ago, transforming the earlier established ancestry.
4. Community sizes in ancient California were small relative to most other areas of the Americas, and Channel Islands people drew from smaller pools of potential mates than those on the mainland.
5. No genetic interaction between Polynesia and California is detectable.
6. Ancient Californians and Mexicans harbored ancestry similar to an individual from the Clovis culture, but ancient Mexicans also have distinct ancestry deriving from a lineage that split earlier.
