# Supplementary material for: Genetic Continuity and Change Among the Indigenous Peoples of California
Source: Nature. Author manuscript; Available in PMC 2024 Feb 16. (PMC10872549; doi:10.1038/s41586-023-06771-5)
Supplement: Supplementary Data File [file NIHMS1941388-supplement-Supplementary_Data_File.pdf]

Genetic Continuity and Change Among the Indigenous Peoples of California

Frequently Asked Questions Summary Sheet

INTRODUCTION

What is ancient DNA?

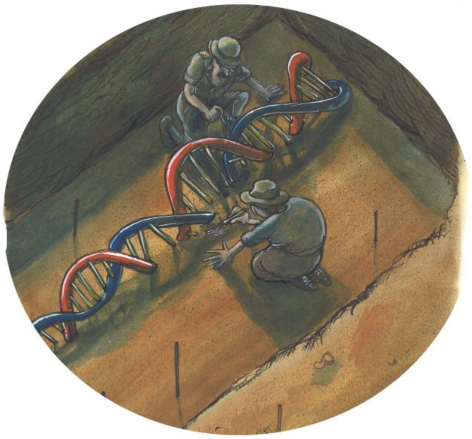

Image: Copyright Martin Rowson; https://www.eurozine.com/unlocking-ancient-code/

Ancient DNA is the remnant molecules of ***DNA (deoxyribonucleic acid)*** that can be recovered from plants, animals, and people who lived hundreds or thousands of years ago. Scientists use a variety of techniques to collect, analyze, and interpret ancient DNA data. When using ancient DNA to study people, scientists can learn about biological connections between modern groups and their ancestors, how similar or different ancient people were to each other and the people who lived near them, and what kind of genetic adaptations they had that helped them survive in their environment. The study of ancient DNA has expanded rapidly since around 2010 as technological improvements have allowed scientists to work more cost effectively and efficiently and collect ancient DNA from bone that is poorly preserved.

How does ancient DNA differ from modern DNA?

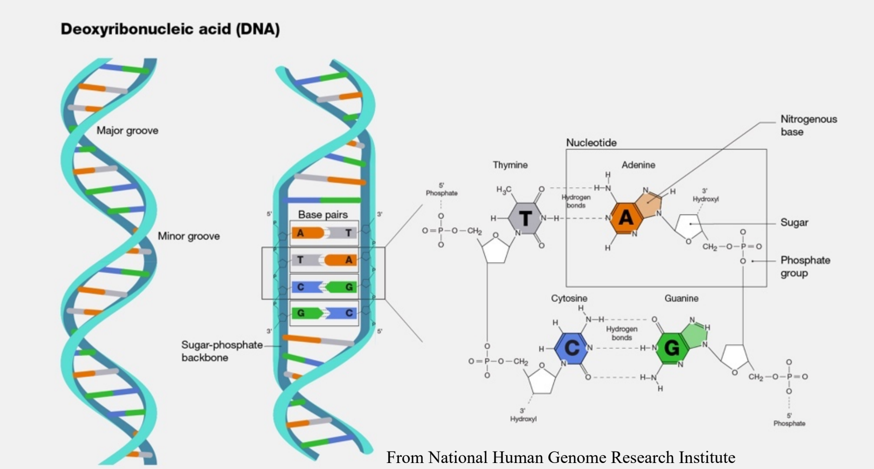

From National Human Genome Research Institute
https://www.genome.gov/genetics-glossary/Deoxyribonucleic-Acid

DNA is the molecule in all living organisms that contains hereditary information with instructions that an organism needs to develop, survive, and reproduce. While ancient DNA and modern DNA are inherently the same—they are molecules arranged in the form of a spiraling ladder, with side pieces (‘backbone’) formed by sugar and phosphate molecules and ladder rungs comprising the repeating chemical bases of adenine (A), thymine (T), cytosine (C), and guanine (G) paired in a specific way (A with T and C with G)—the primary difference between the two is their condition. In living organisms, DNA is a complete chain inside the nucleus of each cell; in humans, this chain is around 3.2 billion ***base pairs*** long. However, the

moment an organism dies, their DNA stops replicating and repairing errors, and instead begins degrading. Therefore, ancient DNA consists of bits and pieces of the DNA chain, sometimes only a few dozen (or even fewer) bases long. In addition to being fragmented, the repair processes that maintained the exact sequence of the base pairs during life no longer function. Because of this, the base pairs are often modified chemically, and sometimes these modified bases can be read incorrectly. For example, a "C" might be read as a "T" after the chemical structure of the base is altered by exposure to water. DNA damage accumulates - so the longer an organism has been dead, the more damage there is likely to be in its DNA. Damage to the DNA molecule also occurs at a rate that depends on environmental factors - researchers now know that hot and humid conditions oftentimes lead to poor DNA preservation, whereas cool, dry, and stable conditions often result in better preservation.

How is ancient DNA used to study the past and how groups of people interacted with one another?

You probably have heard that all modern humans are at least 99.9% genetically identical to one another. This is true! Yet it is also true that there is variation between individuals and groups of people. This variation is due to ***mutations*** that occur in some people's genomes that get passed on to their offspring. Most of these mutations have no effect, but some have led to the evolution of traits that help us live in different environments (for example, the ability to digest lactose as adults was very advantageous for people who practiced animal husbandry because they were able to obtain extra nutrition from dairy sources). A ***population*** is a group of individuals that share some specific traits, most often because people that are in close proximity are more likely to mate and pass on mutations to each other than people who are further apart.

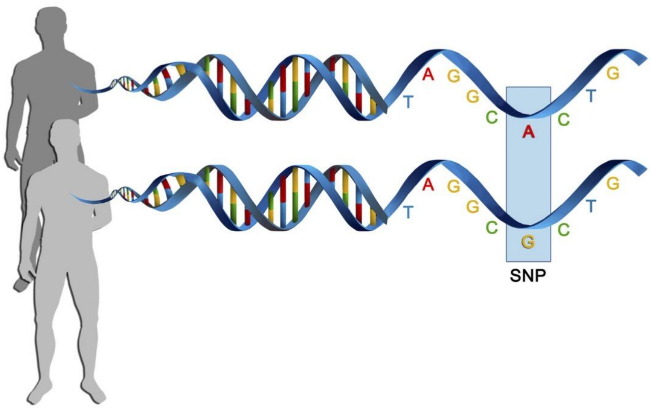

https://www.genengnews.com/topics/omics/study-finds-genetic-basis-of-common-diseases-may-span-tens-of-thousands-of-snps/

Because most of the human genome is shared by all people and therefore not informative about variation, ancient DNA researchers focus on ***Single Nucleotide Polymorphisms*** (**SNPs**, pronounced "snips"), parts of the genome with high variability. These SNPs are single base pairs where any one person can have any one of the possible chemical bases of A, T, C, or G. These SNPs can help explain seen and unseen human variation, such as why some populations are able to drink milk or some have a propensity for sickle-cell disease. Examining large numbers of these SNPs that are distributed throughout the 3.2 billion base pairs of our ***nuclear genome*** can help researchers to quantify differences between people and populations. In this study, researchers used a set of 1.24 million SNPs to study variation in ancient Southern and Central California and Mexican groups.

<u>**METHODS**</u>

How do we study ancient DNA?

Scientists follow an established process to generate ancient DNA data from ancient materials. This process typically involves using a small amount of ancient bone to extract and sequence DNA to learn about the genetic makeup of past peoples.

In popular media, such as television crime dramas, DNA analysis is portrayed as something that is easy to do and rapidly produces accurate results. This is not how ancient DNA research (or any genetic research) is conducted. It takes researchers months if not years in the lab to determine the DNA base pair sequence from the samples obtained, and then even more time to analyze the data and publish it in a report or scientific journal.

Most of this work takes place inside highly specialized facilities called "clean rooms," which help scientists to make sure they are only studying authentic ancient DNA and not contamination from modern DNA. In these clean rooms, trained technicians wear full body suits, facemasks, hairnets, shoe covers, and two pairs of gloves, all to prevent their own DNA from getting mixed up with that of the ancient individual they wish to study. They perform their work under fume hoods and use UV irradiation when the rooms are unoccupied to destroy DNA molecules that may have escaped into the air of the room.

The process of acquiring aDNA from ancient from bone typically involves the following steps:

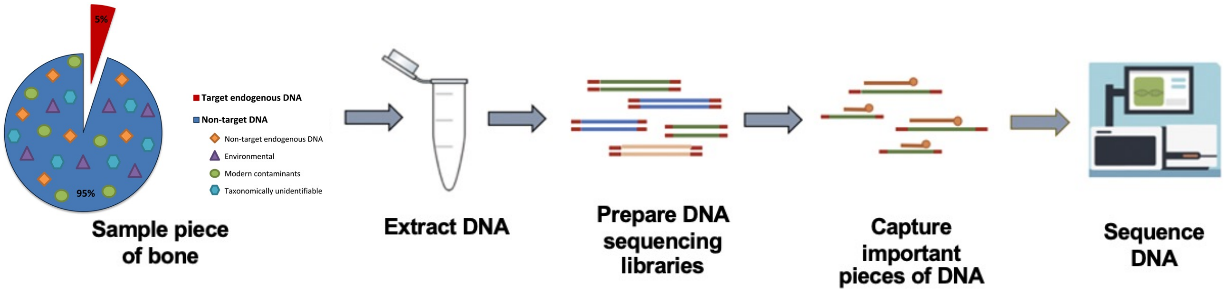

1. A small piece of biological material is selected for ancient DNA analysis. The primary method for acquiring ancient DNA from osteological material is with finely ground powder, particularly from the ***petrous bone***. Ancient DNA researchers continue to refine methods so that damage is minimized. Currently, only 37 milligrams (not much more than a pencil tip) of powder are needed to generate ancient DNA from a skeletal element.
2. DNA molecules are then ***extracted*** from the powder. To extract DNA, the powder is combined with an extraction buffer, which is a watery substance that helps to release DNA into the solution.
3. Once the DNA is recovered, technicians manipulate DNA sequences to create ***DNA libraries.*** One of the most important parts of the process is to attach a special short adapter (a sequence of As, Ts, Cs, and Gs that is known to the researcher) to the ancient DNA molecules. This allows the researchers to know that the molecules came from the bone material of a particular individual. Library preparation is necessary so that the DNA is visible to the ***sequencer*** (below).

4. Sometimes after the DNA sequences are manipulated, lab technicians ***capture*** the most important ***SNPs*** that can help inform about the ancient individual. Capture increases the amount of useful DNA and makes the sequencing process is more efficient. In other cases, the technicians skip this capture step and move right to sequencing; the benefit of skipping capture is that much more data are generated, enabling researchers to explore parts of the genome that we might not have captured if that step had been used.
5. The last step in this process is to analyze the DNA with a ***sequencer***. A sequencer is a specialized machine that "reads" the letters of the DNA sequence (the series of As, Ts, Cs, and Gs) in a library and determines their order.

STUDY OF ANCIENT SOUTH AND CENTRAL CALIFORNIAN AND NORTHERN MEXICAN POPULATIONS

Project Background

This project came together through the collaboration of Indigenous communities and researchers, geneticists, and archaeologists. The project examines ancient DNA from people who lived hundreds or thousands of years ago in what today is California and Northwest Mexico. We know people lived and interacted with one another over millennia. However, the extent of interaction between different groups and biological connections to one another is something archaeologists have debated for decades. Additionally, ancient DNA has not previously been used to help understand how and why California had so many diverse languages prior to European colonization. We analyzed 79 ancient Californians and 40 ancient Mexicans to explore these questions.

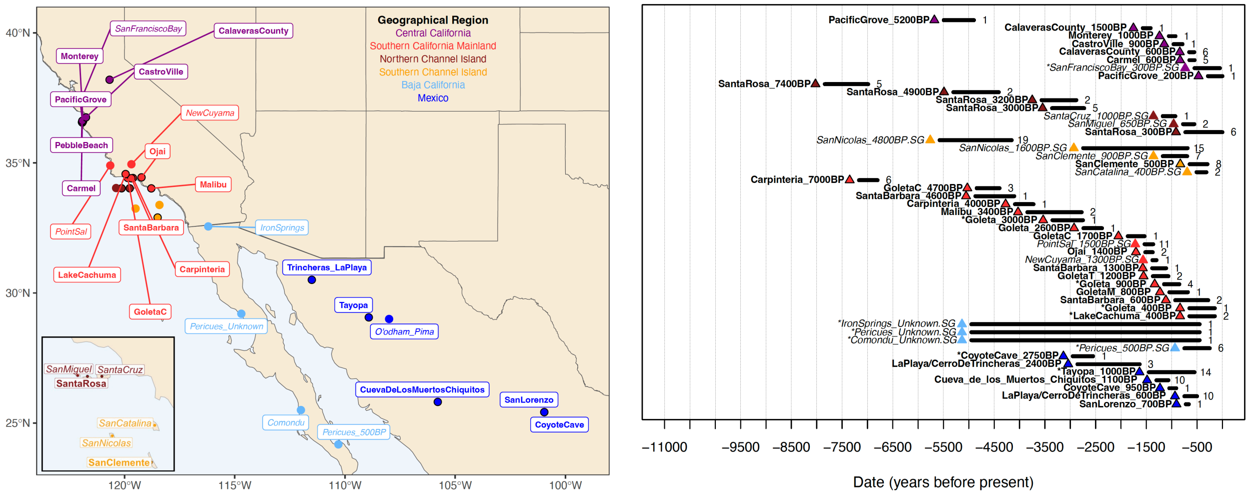

Map with location of archaeological sites examined (left) and time range and number of individuals from each site (right).

What can researchers learn from studying DNA of the people who lived in ancient California and Mexico?

Researchers used ancient DNA to learn much about the lives of the people who lived in California and northern Mexico hundreds to thousands of years ago. Ancient DNA provided insights about the individuals, such as their molecular sex, if they were related to any other individuals analyzed, their

mtDNA haplogroups (passed from mothers to their children), and about the ancient individuals' genetic make-up. By comparing populations, researchers learned if and how different groups interacted with one another through time, and tested hypotheses about these ancient groups, such as if there was a migration of people from Mexico into California associated with the spread of maize farming about 4000 years ago. Researchers also used population-level data to make population size estimates for the various groups in the study.

In addition to ancient DNA, some of the ancient material in this study was sampled to produce radiocarbon dates. ***Radiocarbon dating*** allowed researchers to determine the time period when an ancient individual lived. The methods used for radiocarbon dating also allow researchers to examine ancient diets, for example if maize was a significant part of their diet, how much seafood they ate, etc.

Who participated in this research?

We received scientific support from the Santa Barbara Museum of Natural History, the Peabody Museum of Anthropology and Ethnology, Harvard University, and from archaeological excavations conducted for proposed development projects in which State-designated Most Likely Descendants had approved ancient DNA testing. Ancient Mexican samples were obtained from the American Museum of Natural History in New York, the Peabody Museum, the University of Nevada Las Vegas, and Museo Nacional de Antropología in Mexico.

What will happen to the bones now that the study is completed?

The vast majority of biological material used during this study has already been repatriated to the proper tribal groups in California. For ancient Mexican individuals still in US museums, the researchers have shared information with Mexico's Instituto Nacional de Antropología e Historia and they are determining how best to proceed with repatriation efforts.

How were the data analyzed?

Researchers used a number of different statistical methods to learn about the individuals in this study and their associated populations.

F_3 statistics were used to quantify how closely related the ancient Californian and Mexican populations were to each other and other ancient and modern populations. These statistics require researchers to sort the samples into groups; the groups are then tested against each other and an "outgroup" (Mbuti) that should be equally elated to both test groups. Researchers can then use the results to construct "trees" that show which groups are more closely related (figure below).

F_4 statistics are similar to f_3 statistics, except an extra "test" population is added. This allows researchers to see if two populations form a "clade," meaning they are more closely related to each other than either is to the test population. For example, f_4 statistics revealed that the early Santa Rosa individuals (approximately 7400 years old) formed a clade with all later Santa Rosa individuals more recent than 7400 BP when tested against almost all other populations outside of California. An exception to this is that

when younger Santa Rosa groups were tested against some recent Mexican groups, such as those from a site in Sonora dating to approximately 1100 years ago, the younger Santa Rosa groups had more similarity to the Mexican groups than the older Santa Rosa groups. This suggests that for most of their history the people who lived on Santa Rosa Island reproduced primarily with people from the Island (and nearby on the mainland); however, there was some gene flow from Mexico more recently in time.

qpAdm is another form of analysis that uses f_4 statistics to examine the proportion of ancestry an individual or group might have from different groups. For example, qpAdm analysis found that approximately 20% of the ancestry of individuals from Santa Rosa dating to about 4900 years ago is most similar to Mexican populations (the majority ~80% of their ancestry is most similar to Santa Rosa individuals from about 7400 years ago). Researchers can thus use this method to study how ancestry proportions changed though time.

ADMIXTURE analysis compares all the ancient (and modern) individuals in the study to each other to determine their ancestry profile. Researchers can set the analysis to determine a specific number of components; in this study 2-7 different components were used. Researchers can then compare the ancestry components of different groups to examine their similarities and differences.

Runs of Homozygosity are parts of the genome that repeat themselves. Researchers can measure and count runs of homozygosity to explore the degree of relatedness between a person's first few generations of lineal ancestors (e.g., parents, grandparents, and great parents) and use this data to create population size estimates. For example, a person having many short runs of homozygosity indicates that their immediate ancestors lived in a small group in which many people were related to each other to some degree.

What are the key results?

Using the methods above, researchers have made several exciting discoveries about the ancient people who lived in Southern and Central California and Northern Mexico. Population-level analysis show that in general the individuals in the study cluster together based on geography and that there was long-term genetic continuity in Southern and Central California (at least 7,400 years in Southern California and 5,200 years in Central California).

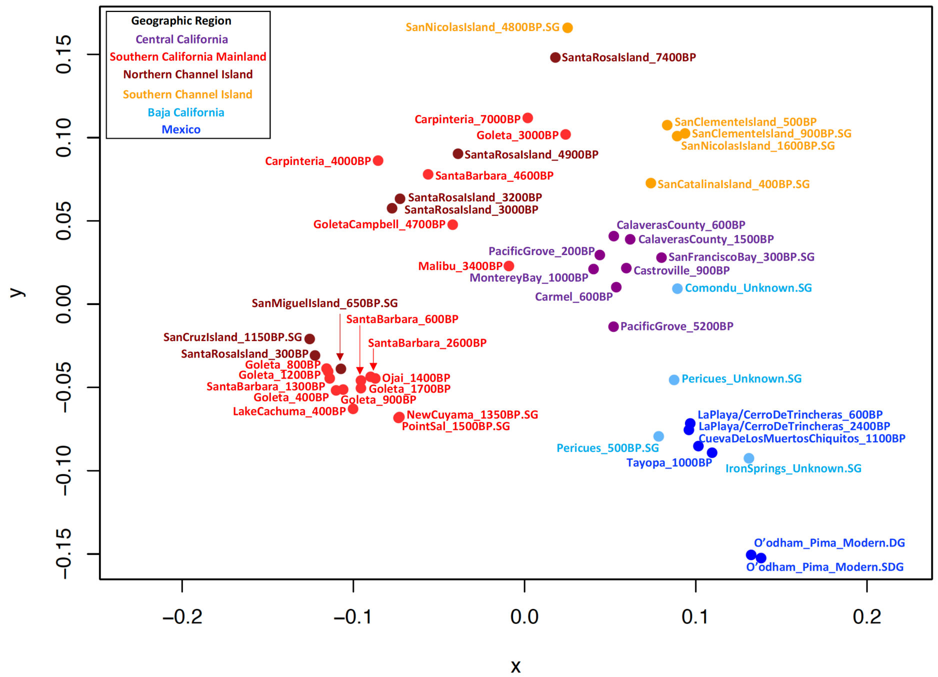

Multi-Dimensional Scaling plot of groups in study created using a matrix of inverted outgroup-f_3 statistics. This shows that groups in closest geographic proximity tend to cluster with one another.

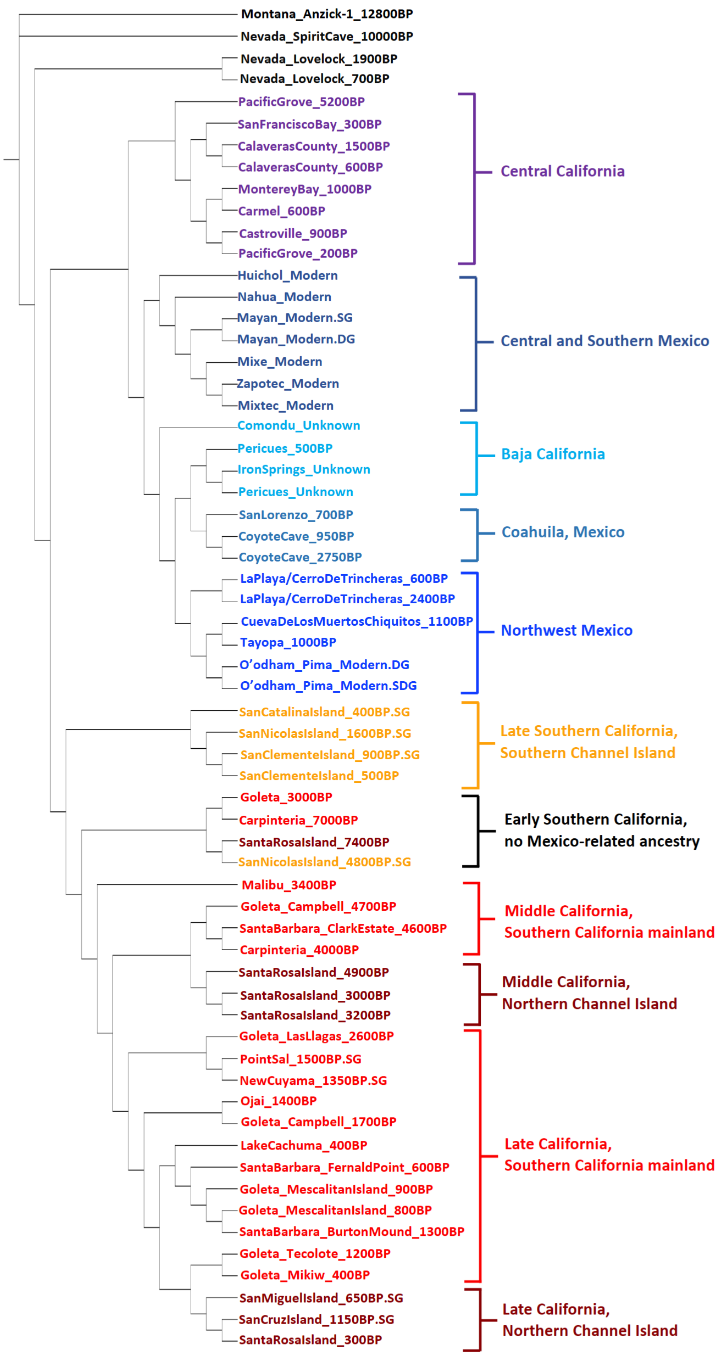

Neighbor-joining tree created using a matrix of inverted outgroup-f_3 statistics. Like the MDS plot above, this shows that groups in closest geographic proximity tend to cluster with one another.

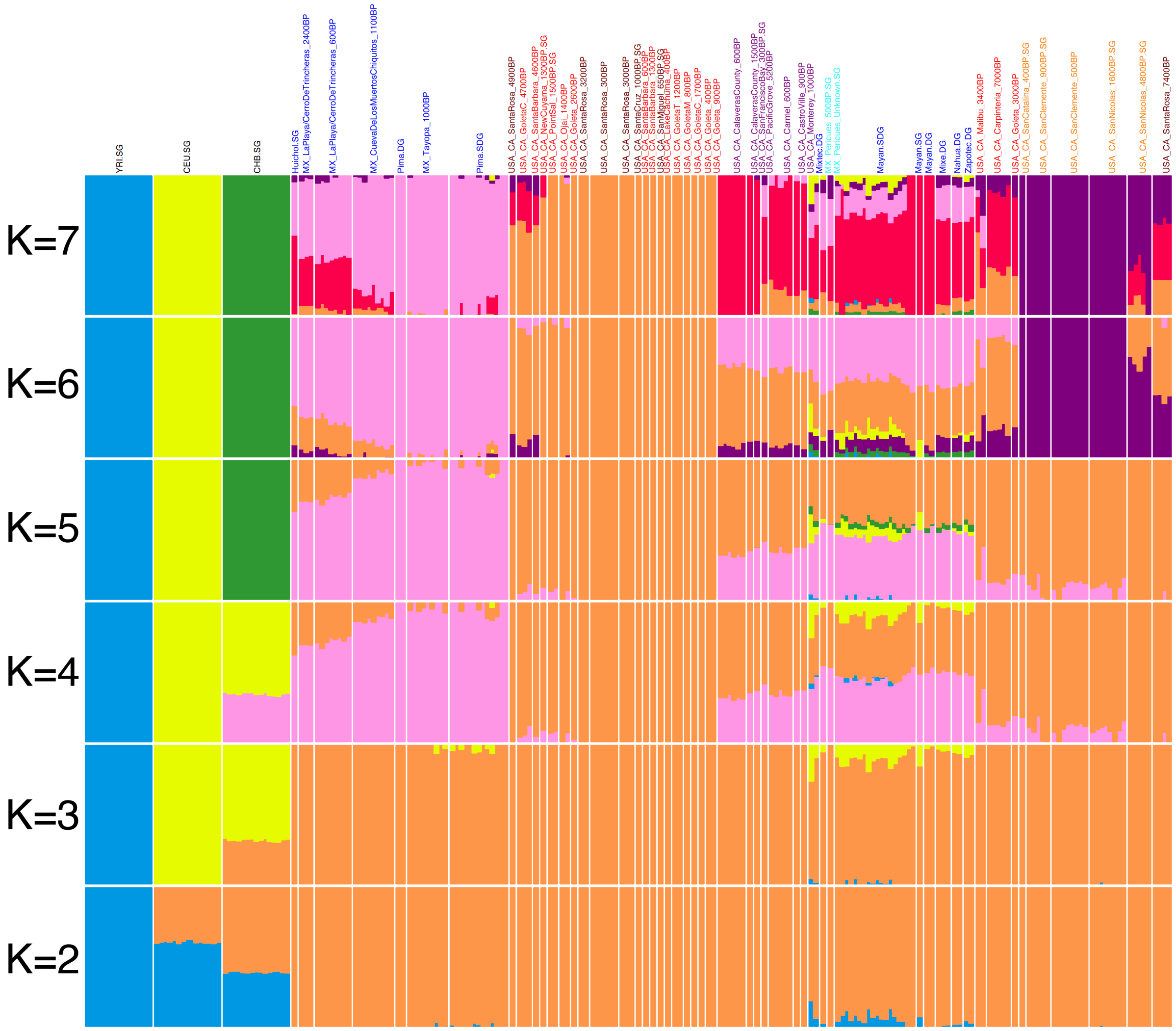

ADMIXTURE plot at different number of ancestry components. Different colors represent different ancestry components.

This project has also demonstrated that gene flow related to people from Northern Mexico moved into Southern and Central California by at least 5200 years ago. This is an important discovery, because archaeologists and anthropologists have long-debated whether there was an expansion of Mexican-related ancestry with the spread of maize, which occurred around 4000 years ago. This study suggests that gene flow from south to north predates the spread of maize agriculture, and that it increased over time. The ancestry is related to groups that speak what are called "Uto-Aztecan" languages. Some groups that speak "Uto-Aztecan" languages today are the O'odham, Tongva (Gabrieliños), Tarahumara, Yaqui, and Luiseño (Payómkawichum).

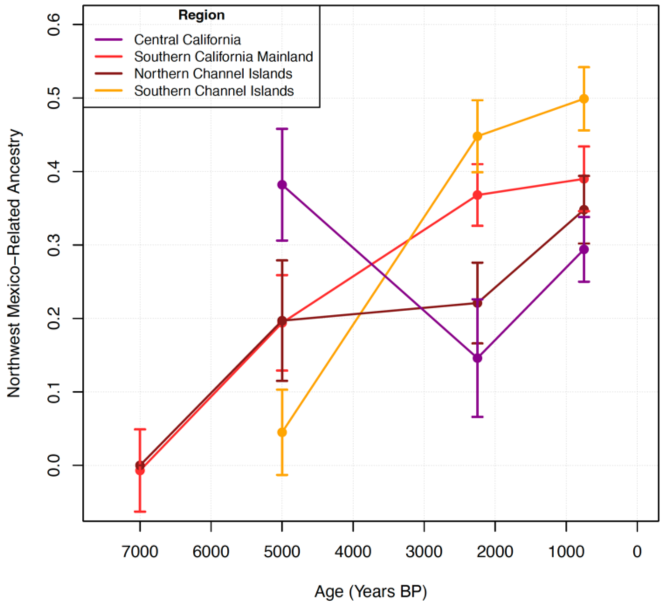

Northwest Mexico-related ancestry at different regions over time, from qpAdm. Each data point represents average Mexico_LaPlaya/CerroTrincheras_600 BP-related ancestry in a bin of time (8000-6000 BP, 6000-4000 BP, 4000-1500 BP, and <1500 BP). Bars represent ±1 standard error.

The study also showed that people from the coast and inland of Central California mixed with each other over the past 5,000 years. The researchers, however, found no evidence of genetic interaction between Polynesia and California before 200 years ago.

Researchers were also able to use runs of homozygosity to determine the group size of individuals in the study and found that populations from the Californian Channel Islands were small (reflecting the small size of the islands), and a similar size as populations in ancient Patagonia, the Caribbean islands before they had agriculture, and the islands Guam and Saipan. Mexico had a population size approximately the size of ancient Peru and Caribbean after agriculture, while mainland California had a size in between those of the Channel Islands and Mexico. Note that an effective population size is not the same as an actual population size. An effective population size is a genetic term for the population size if there were complete randomness in genetic mixing, and in general, the actual population size is sometimes 5x larger than the effective population size.

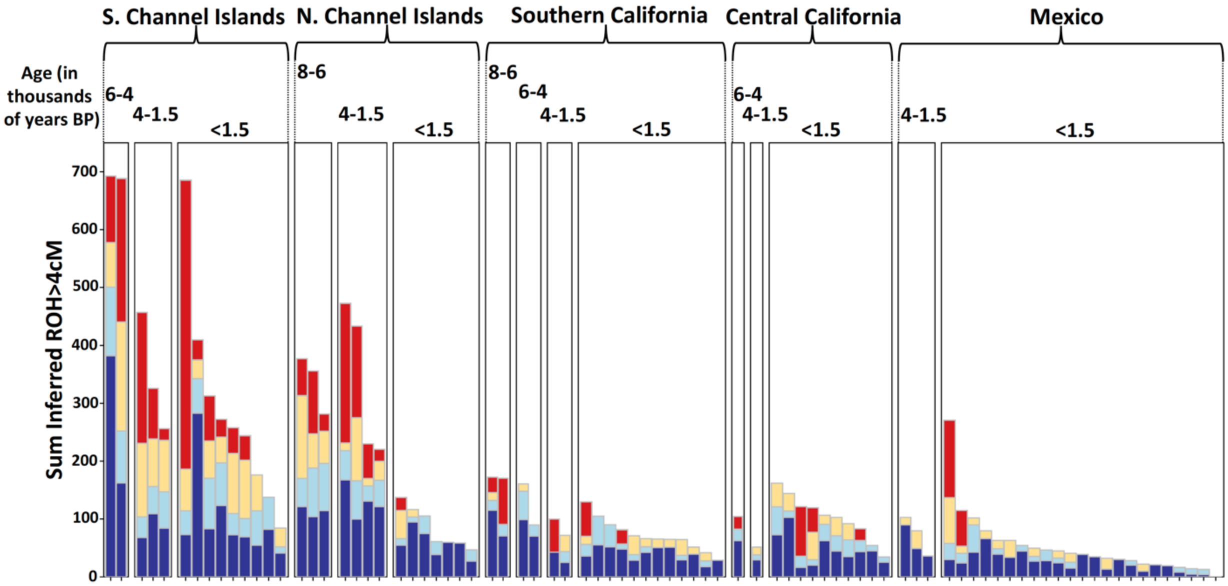

Above— Runs of Homozygosity (ROH) in all ancient Californians and Mexicans with sufficient sequencing coverage. Dark blue indicates sum ROH of 4-8cM fragments; light blue 8-12 cM; tan 12-20 cM; and red 20-300 cM fragments. Individuals with large red segments had ancestors who were closely related. Different regions grouped by age. Left—Effective population size estimates obtained by HapROH with point estimates and one standard errors. Error bars represent ±1 standard error.

Group	Pop. Size
Patagonia <1500BP	171 ± 7
S. Channel Islands <1600 BP	175 ± 13
Caribbean_Archaic <3200 BP	232 ± 8
Venezuela_Ceramic 2000-3000 BP	274 ± 25
Guam <800 BP	333 ± 11
Saipan <800 BP	375 ± 16
N. Channel Islands <1500 BP	388 ± 42
Central California <1600 BP	418 ± 39
S. California Mainland <1500 BP	519 ± 51
Bolivia <1700 BP	663 ± 62
Caribbean_Ceramic <1700 BP	681 ± 21
Peru <1800 BP	817 ± 51
Mexico <1600 BP	839 ± 74

Another reason that this study is important is that it is one of the first to publish ancient DNA data from northern Mexico. These data not only allowed researchers to examine how and when gene flow from the south arrived in Southern and Central California, but will allow researchers to continue researching how these groups relate to other ancient and modern groups.

RESEARCH IMPACTS

What if the results of the genetic analysis are different from what we know to be true from our oral history/traditional knowledge?

The current study is primarily aimed at adding to scientific knowledge about the interactions of Native American populations and understanding of archaeological cultures. Scientific knowledge is only one way of knowing about the world, and Indigenous ways of knowing are also important ways to approach the world. Different cultures will have different ways in which they approach the balance and integration of scientific knowledge and traditional, ancestral ways of knowing, and it is important to have discussions

within one's own cultural group about how best to approach scientific results while also promoting cultural revival and resilience.

It is also important to note that genetics does not equal identity or culture. Identity and culture are often based on group and individual dynamics, including cultural practices and social relationships. Cultural groups may define an individual's group identity in a manner that has little to do with that individual's genetics. Thus, genetic results should not be used to invalidate one's cultural identity.

What might this research mean for me as an Indigenous person of California or Mexico?

This research is about the ancient people who lived in California and Mexico. It is a scientific/genetic and archaeological project about people who lived hundreds and thousands of years ago. This research can add another perspective to what is already known about the interactions Indigenous people in the area had with other Indigenous groups from other areas, and for some people, it might enrich their understanding of themselves and how they are connected with other Indigenous groups.

GLOSSARY OF TERMS

Base/base pair– DNA bases, also known as nucleotides (see below), are the "steps" on the DNA ladder.

Capture– Laboratory process of selecting and preparing informative SNPs for sequencing.

DNA– DNA is a molecule that contains the genetic instructions for growth and development of all organisms. The DNA molecule is made up of smaller molecules called nucleotides. There are four nucleotides called Adenine (A), Thymine (T), Cytosine (C), Guanine (G) that make up the DNA molecule.

DNA libraries– A DNA library is a collection of smaller DNA fragments that make up a full genome of an organism.

Extract–Solution containing all DNA molecules from dissolved bone powder.

Genome– All of the DNA within an organism. This includes both nuclear DNA and mitochondrial DNA.

Haplogroups– Haplogroups are used to group individuals based on their genes, which could inform about a group's origin. To be more specific, haplogroups are a set of genes or mutations in genes that get passed on through generations. These genes can be specific to certain regions in the world and so they can be indicative of where a person came from.

Mitochondrial DNA (mtDNA)– This DNA comes from the mitochondria within a cell and is distinct from nuclear DNA. This is important because mtDNA is passed from a mother to their offspring. The

mitochondrial genome consists of about 16,000 base pairs and is much smaller than the nuclear genome.

Nuclear Genome– This refers to the DNA located inside the nucleus of the cell. In humans, the nuclear genome is made up of over 3 billion base pairs.

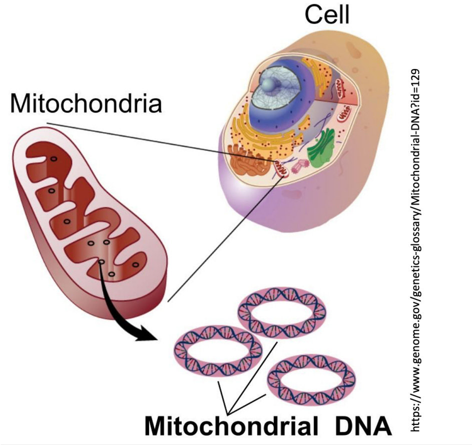

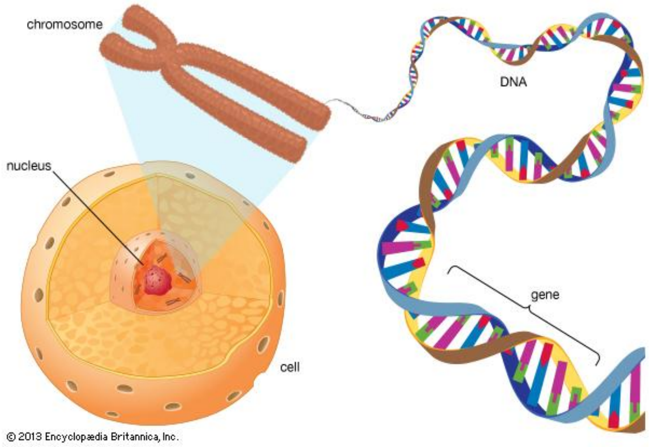

Petrous– The petrous bone is part of the temporal bone. It currently is the preferred bone for ancient DNA analysis as studies have shown it is the part of the body that preserves the most aDNA.

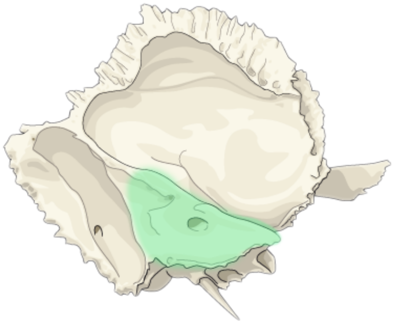

Radiocarbon analysis– Also known as radiocarbon dating, is used by archaeologists to determine how old organic material is in bone, wood, or other preserved organic material. The method involves measuring Carbon 14 (C14) isotopes to calculate the date of a once living organism.

Sequencing–Process of determining the order of A's, T's, C's, and G's in a sample.

Single Nucleotide Polymorphisms (SNPs)– Variations of a single base pair in DNA among different organisms. These variations give rise to different characteristics among organisms.

Uniparental lineages- DNA segments that are passed only from mother to offspring or father to sons and can therefore be used to study particular lineages. For males, the Y chromosome is passed only from father to son(s). Females pass mitochondrial DNA to all of their offspring, but males do not. Unique

mutations in mtDNA and Y chromosome DNA that have accrued through time allow researchers to identify and trace specific lineages (aka **haplogroups**) through time.
