# Supplementary material for: Genetic Continuity and Change Among the Indigenous Peoples of California
Source: Nature. Author manuscript; Available in PMC 2024 Feb 16. (PMC10872549; doi:10.1038/s41586-023-06771-5)
Supplement: Supplementary Information Guide [file NIHMS1941388-supplement-Supplementary_Information_Guide.pdf]

Supplementary Information Guide

Genetic Continuity and Change Among the Indigenous Peoples of California

NOTE ON LOCAL GENETIC CONTINUITY IN MEXICO……………………...……...2

ARCHAEOLOGICAL SITE SUMMARIES………………………..…………………..3-13

- NUMBER OF INDIVIDUALS PER SITE………………………………………………………...3
- SAN CLEMENTE ISLAND (CALIFORNIA)………………………………………………………4
- SANTA ROSA ISLAND (CALIFORNIA)…………………………………………………………4-5
- MALIBU (CALIFORNIA)……………………………………………………………………………5
- OJAI (CALIFORNIA)……………………………………………………………………...…5
- CARPINTERIA (CALIFORNIA)……………………………………………………………..……..6
- SANTA BARBARA (CALIFORNIA)……………………………………………………………......7
- GOLETA (CALIFORNIA)…………………………………………………………………….....6-7
- LAKE CACHUMA (CALIFORNIA)……………………………………………………………...…7
- CARMEL (CALIFORNIA)……………………………………………………………………...….8
- PEBBLE BEACH (CALIFORNIA)……………………………………………………………...…...8
- MONTEREY (CALIFORNIA)……………………………………………………...……….8
- PACIFIC GROVE (CALIFORNIA)…………………………………………………………….....8-9
- CASTROVILLE (CALIFORNIA)………………………………………………………………..…..9
- CALAVERAS COUNTY (CALIFORNIA)…………………………………………………...…...9
- CERRO DE TRINCHERAS, SONORA (MEXICO)……………………………………..….....9-10
- TAYOPA, SAHUARIPA, SONORA (MEXICO)……………………………………….………10-11
- CUEVA DE LOS MUERTOS CHIQUITOS (MEXICO)……………………………………..…..11-12
- SAN LORENZO CAVE AND COYOTE CAVE, COAHUILA (MEXICO)……………………….12-13

REFERENCES………………………...…………………………………………………14-15

Supplementary Data Files

As separate Excel (.xlsx) files and 1 pdf file

Supplementary Data File 1
(A) Meta-data about individuals newly sequenced or previously published from California or Northwest Mexico. **(B)** Technical information on each ancient DNA library built for all samples newly sequenced in this study as well as failed samples. **(C)** Meta-data of individuals previously published from other world regions that we co-analyzed with those from California and Northwest Mexico.

Supplementary Data File 2
qpWave, showing genetic homogeneity within groups.

Supplementary Data File 3
F_{ST} between different groups.

Supplementary Data File 4
f_4 results as tests of admixture and qpgraph worst fit Z-scores.

Supplementary Data File 5
qpAdm estimates of ancestry.

Supplementary Data File 6
qpWave analyses of migration into the Andes.

Supplementary Data File 7
Inferred Ne from ROH.

Supplementary Data File 8
Frequently Asked Questions (FAQ) document prepared for Indigenous community members and others about the paper.
