# Supplementary material for: Genetic Continuity and Change Among the Indigenous Peoples of California
Source: Nature. Author manuscript; Available in PMC 2024 Feb 16. (PMC10872549; doi:10.1038/s41586-023-06771-5)
Supplement: Supplementary Information [file NIHMS1941388-supplement-Supplementary_Information.pdf]

Supplementary Information

Genetic Continuity and Change Among the Indigenous Peoples of California

Local Genetic Continuity in Mexico:

The Tradición Trincheras (Trench) culture consisted of terraced agricultural villages built on hills in the Sonoran Desert. Archaeologists have debated the degree to which Tradición Trincheras people interacted with their neighbors to the north (Hohokam) and east (Casas Grandes), although at least some Hohokam incursion did occur [1,2]. Approximately 600 years ago the Tradición Trincheras declined and by the time the Spanish arrived the local material culture bears clear relationship to modern O'odham. As noted, when we compared the *LaPlaya/CerroDeTrincheras_600 BP* individuals to the *LaPlaya/CerroDeTrincheras_2400 BP* individuals, no significant differences were observed (Supplementary Data File 4), consistent with local genetic continuity. Comparisons with other Mexico individuals only showed genetic affinities correlating with geography (*O'odham_Pima_modern* and *Cueva de los Muertos Chiquitos_1100 BP* showed greater affinity for *Tayopa_1000 BP*, which are closer to them geographically). More data from individuals who lived during and after the transition to the O'odham culture would provide further insight.

Details about analyzed individuals:

We generated new genome-wide data from skeletal remains of 79 ancient individuals from California and 40 ancient individuals from Mexico:

California:

- San Clemente Island: 8
- Santa Rosa Island: 20
- Malibu: 4
- Ojai: 2
- Carpinteria: 7
- Santa Barbara: 5
- Goleta: 14
- Lake Cachuma: 2
- Carmel: 5
- Pebble Beach: 1
- Monterey: 1
- Pacific Grove: 2
- Castroville: 1
- Calaveras County: 7

Mexico:

- Cueva de los Muertos Chiquitos: 10
- Coyote Cave: 2
- San Lorenzo: 1
- Tayopa: 14
- La Playa/Cerro De Trincheras sites: 13

Description of Archaeological Sites:

A tabulation of all information obtained for genetic or radiocarbon dating, including genetic sex and carbon and nitrogen isotope data, is presented in Supplementary Data File 1. When choosing ancient individuals from whom to obtain genetic data, we adopted an iterative approach, focusing on obtaining data from individuals from many different areas and different time periods to understand the geographic extent of the different ancestry profiles over time. We then increased the number of individuals sequenced as needed to ensure that our findings were statistically robust, while also minimizing the number of individuals sequenced out of respect for Indigenous ancestors. All relevant ancient skeletons at the Santa

Barbara Museum of Natural History, which is the source of many of the ancient individuals in this study, have now been repatriated and/or reburied.

<u>San Clemente Island (California):</u> 645-295 calBP

- I0748: 950-700 BP
- I0750: 950-700 BP
- I0751: 635-295 calBP (marine corrected)): 1157-1225 calCE (865±20 BP, PSUAMS-6495/UCIAMS-158549)
- I0752: 950-700 BP
- I0755: 950-700 BP
- I0756: 950-700 BP
- I0758: 950-700 BP
- I0760: 645-305 calBP (marine corrected): 1053-1223 calCE (875±20 BP, PSUAMS-6496/UCIAMS-158550)

San Clemente Island is the southernmost of California's Channel Islands, 147 km^2 in size and located 79 km from the nearest point on the mainland. The seven individuals reported here resulted from Paul Schumacher's archaeological investigations on the island in 1877. No specific site provenience was recorded for the remains in Schumacher's collection; however, his correspondence indicates that he was camped on the north end of the island, indicating he likely excavated a site close by. The individuals included in our study came from two genetically male and six genetically female individuals.

<u>Santa Rosa Island (California):</u> 7840-0 calBP

<u>CA-SRI-3A</u>

- I2709: 5305-4645 calBP (marine corrected): 3621-3366 calBCE (4680±40 BP, Poz-83502)
- I2713: 5025-4410 calBP (marine corrected): 3339-2932 calBCE (4450±40 BP, Poz-83505)
- I2710: 7840-7315 calBP (marine corrected): 5982-5755 calBCE (6990±40 BP, Poz-83503)
- I2711: 7425-6985 calBP (marine corrected): 5477-5372 calBCE (6455±25 BP, PSUAMS-2153)
- I2712: 7700-7170 calBP (marine corrected): 5835-5667 calBCE (6865±35 BP, Poz-83504)
- I11557: 7665-7100 calBP (marine corrected): 5726-5631 calBCE (6785±30 BP, PSUAMS-5726)
- I11558: 7655-7240 calBP (marine corrected): 5724-5631 calBCE (6780±30 BP, PSUAMS-5727)

<u>CA-SRI-5A</u>

- I11968: 3565-2885 calBP (marine corrected): 1614-1461 calBCE (3270±25 BP, PSUAMS-5768)
- I11969: 3535-2880 calBP (marine corrected): 1607-1450 calBCE (3255±20 BP, PSUAMS-5769)

<u>CA-SRI-41A</u>

- I3562: 3400-3000 BP
- I3563: 3400-3000 BP
- I3564: 3360-2725 calBP (marine corrected): 1447-1298 calBCE (3120±25 BP, PSUAMS-2116)
- I3565: 3140-2745 calBP (marine corrected): 1260-1055 calBCE (2955±25 BP, PSUAMS-2073)
- I2708: 3245-2855 calBP (marine corrected): 1391-1130 calBCE (3020±30 BP, Poz-83479)

<u>CA-SRI-2A</u>

- I11967: 730-335 calBP (marine corrected): 993-1027 calCE (1030±15 BP, PSUAMS-5731)

<u>CA-SRI-2B</u>

- I2703: 700-500 BP
- I2704: 260-0 calBP (marine corrected): 1301-1410 calCE (595±30 BP, Poz-83443)
- I2705: 505-80 calBP (marine corrected): 1262-1387 calCE (710±30 BP, Poz-83444)
- I2706: 420-0 calBP (marine corrected): 1277-1391 calCE (675±30 BP, Poz-83445)
- I2707: 700-500 BP

Wima (Santa Rosa Island) is the second largest of the Northern Channel Islands, 215 km^2 in size, located approximately 41.6 km off the coast from Santa Barbara, California. Twenty individuals from four archaeological sites were included in this study. Seven individuals were from CA-SRI-3A, excavated by Phil C. Orr of the Santa Barbara Museum of Natural History in 1949-1951, derived from two superimposed occupations at this location. The majority of interments, two genetically male and three genetically female, represent the most ancient cemetery assemblage, dating to between 5982-5372 calBCE. Two later interments, one genetically male and one genetically female, were intrusive into the earlier cemetery and dated between 3621-2932 calBCE. Two individuals came from CA-SRI-5A, investigated by Orr in 1949. These two individuals, one genetically male and one genetically female, lived between 1614-1450 calBCE. Five individuals came from CA-SRI-41A, excavated by Orr in 1951 and 1957. These burials, all genetically female interments, dated between 1447-1055 calBCE. Samples from six individuals were from CA-SRI-2, excavated by Orr in 1950 and 1952. CA-SRI-2 has been tentatively correlated with the ethnohistoric Island Chumash ranchería of either Niaqla or Nimkɨlkɨl [3]. One individual was a genetically female burial from Cemetery A, dated between 993-1027 calCE. Five individuals (three genetically male and two genetically female) whose remains had been reburied in prehistoric times, dated between 1262-1410 CE. For further details regarding these Santa Rosa Island sites, see Orr (1968) [4].

<u>Malibu (California):</u> 3845-2775 calBP

<u>CA-LAN-222</u>

- I14825: 3180-2775 calBP (marine corrected): 1377-1130 calBCE (3005±20 BP, PSUAMS-7672)
- I14826: 3845-3585 calBP (marine corrected): 1890-1749 calBCE (3505±20 BP, PSUAMS-7673)
- I14827: 3950-2950 BP
- I14828: 3950-2950 BP

CA-LAN-222 is located on a bluff overlooking the Pacific Ocean in the city of Malibu, Los Angeles County. The site mostly was destroyed by development in the 1960s. Excavations took place in 2018 by archaeologists from Rincon Consultants as part of mitigation for a utilities upgrade project [5]. Analysis of remains from four prehistoric Chumash burials was undertaken with permission from the Most Likely Descendant designated by the Native American Heritage Commission. The burials reported here consisted of two genetically male and two genetically female individuals.

<u>Ojai (California):</u> 1520-1375 calBP

<u>CA-VEN-61</u>

- I11559: 1520-1375 calBP (marine corrected): 433-571 calCE (1555±20 BP, PSUAMS-5728)
- I11970: 1500-1400 BP

The City of Ojai is located in inland Ventura County, 37 km north of the City of San Buenaventura. CA-VEN-61, was excavated by Phil C. Orr in 1942. Orr reported two cemeteries at this site (Locality 111) [6]. The two samples reported here came from two individuals, male and female, from Cemetery A.

<u>Carpinteria (California):</u> 7165-3725 calBP

CA-SBA-7

- I11290: 7200-7000 BP
- I11291: 7165-6850 calBP (marine corrected): 5216-5045 calBCE (6190±25 BP, PSUAMS-4782)
- I11292: 7200-7000 BP
- I15168: 7160-6800 calBP (marine corrected): 5213-5035 calBCE (6170±25 BP, PSUAMS-7733)
- I15476: 7160-6800 calBP (marine corrected): 5213-5035 calBCE (6170±25 BP, PSUAMS-7728)
- I15477: 7200-7000 BP

CA-SBA-1

- I11261: 4090-3725 calBP (marine corrected): 2191-1982 calBCE (3690±20 BP, PSUAMS-4879)

Carpinteria is a coastal town in southern Santa Barbara County. Seven individuals originated from burials excavated at two sites near Carpinteria and its vicinity. The earliest site is part of CA-SBA-7 and was excavated in 1925 by David Banks Rogers of the Santa Barbara Museum of Natural History. Two genetically male and four genetically female individuals date between 5216-5035 calBCE. One individual came from CA-SBA-1 which was excavated by Phil C. Orr in 1950. This male burial dates to 2191-1982 calBCE. For further information regarding these sites, see Rogers (1929) [7].

<u>Santa Barbara (California):</u> 4870-280 calBP

CA-SBA-20

- I14816: 4950-4750 BP
- I15478: 4870-4105 calBP (marine corrected): 3092-2917 calBCE (4390±25 BP, PSUAMS-7617)

CA-SBA-28

- I11283: 1385-1110 calBP (marine corrected): 432-551 calCE (1570±15 BP, PSUAMS-4896)

CA-SBA-17

- I11262: 930-675 calBP (marine corrected): 896-1020 calCE (1080±15 BP, PSUAMS-4880)
- I11282: 675-280 calBP (marine corrected): 992-1038 calCE (1010±15 BP, PSUAMS-4881)

The City of Santa Barbara is located 15 km west of Carpinteria. Individuals from three sites within the city and its neighboring community of Montecito to the east were analyzed. Two individuals, both genetically male, came from CA-SBA-20, investigated by D. B. Rogers in 1933. A single 14C date indicates these burials dated between 3092-2917 calBCE. One individual came from CA-SBA-28, from skeletal remains encountered during grading at this site in 1973. This individual dated to 432-551 calCE. Two individuals came from CA-SBA-17, which was excavated by Rogers in 1924. The burials from Fernald Point, one genetically male and one genetically female, dated between 896-1038 calCE.

<u>Goleta (California):</u> 4870-150 calBP

CA-SBA-52

- I15481: 4870-4725 BP
- I11288: 4845-4395 calBP (marine corrected): 2918-2885 calBCE (4290±20 BP, PSUAMS-4898)
- I11289: 4845-4535 calBP (marine corrected): 2907-2774 calBCE (4240±20 BP, PSUAMS-4899)
- I11287: 1865-1535 calBP (marine corrected): 41 calBCE - 106 calCE (1990±15 BP, PSUAMS-4897)

CA-SBA-48

- I15479: 3350-2750 BP

CA-SBA-81

- I11296: 2740-2375 calBP (marine corrected): 805-773 calBCE (2590±20 BP, PSUAMS-4900)

CA-SBA-73

- I11294: 1370-1065 calBP (marine corrected): 428-540 calCE (1590±15 BP, PSUAMS-4761)

CA-SBA-72

- I11293: 1250-950 BP

CA-SBA-46A

- I11284: 1045-680 calBP (marine corrected): 905-1270 calCE (1265±20 BP, PSUAMS-13126)
- I21249: 1150-750 BP
- I21250: 1150-750 BP
- I21452: 1150-750 BP

CA-SBA-46C

- I14817: 750-650 BP

CA-SBA-78

- I11295: 650-150 BP

The coastal city of Goleta is located just west of and adjacent to Santa Barbara. Ten of fourteen individuals came from three sites that surrounded the ancient Goleta Slough, and four samples came from sites within 20 km further up coast. The Goleta Slough site with the earliest dates was CA-SBA-52, excavated by David Banks Rogers in 1925. Four individuals, two genetically male and two genetically female, came from his investigations here. Two of these dated to 2918-2774 calBCE, whereas a genetically female burial dated to 41 calBCE-106 calCE. Thus, it would appear that this site was occupied at two different periods of prehistory. A nearby site, CA-SBA-48, was investigated by Phil C. Orr in 1941. Recent work at CA-SBA-48 on the current UC Santa Barbara campus indicates that the earlier component from which Orr excavated a genetically male burial dated between 1385-780 calBCE [8].

A single genetically male burial at CA-SBA-81, excavated by Rogers in 1925, yielded a date of 805-773 calBCE. Two individuals came from Rogers's excavations at CA-SBA-73 and CA-SBA-72 just west of Goleta. The genetically female burial at CA-SBA-73 dated to 428-540 calCE. The genetically male burial at CA-SBA-72 likely dates between 950-1250 CE [9]. Five individuals derived from Orr's excavations at CA-SBA-46[10]. Four of these were from Cemetery A, all genetically male burials. Most of this cemetery dates to 800 to 1200 CE; however, a few burials, including one reported here, appear to date somewhat earlier, 600-900 CE. One genetically female burial came from Cemetery C, which had a period of use from about 1200 to 1300 CE. A single genetically female burial whose skeletal remains were brought to the museum in 1979 came from the site of CA-SBA-78, west of Goleta. CA-SBA-78 was the location of the ethnohistoric Barbareño Chumash ranchería of *Mikiw*. The female burial from *Mikiw* likely dates to the Late Period of Chumash prehistory, 1300-1800 CE.

Lake Cachuma (California): 650-150 BP

CA-SBA-477

- I11285: 650-150 BP
- I11286: 650-150 BP

Lake Cachuma is located along the Santa Ynez River, about 32 km northwest of Santa Barbara. Excavations were conducted in 1952 at CA-SBA-477 prior to the inundation of the reservoir. This site has been identified as the ethnohistoric ranchería of *Teqepsh*. Two genetically male burials most likely date to the Late Period of Santa Barbara prehistory, 1300 to 1800 calCE.

<u>Carmel (California):</u> 650-550 calBP

- I11256: 650-150 BP
- I11257: 650-150 BP
- I11259: 650-150 BP

<u>CA-MNT-1482 (CA-MNT-1913)</u>

- I11551: 650-550 calBP (marine corrected): 1302-1398 calCE (615±15 BP, PSUAMS-5725)

<u>CA-MNT-1489</u>

- I11260: 650-150 BP

Carmel-by-the-Sea is a coastal city located around 4 km southwest of Monterey. The individuals analyzed for this study resulted from archaeological investigations by Gary Breschini and Trudy Haversat. Three of the individuals were identified as being from Carmel at the time they were submitted; however, Breschini passed away before specific provenience was obtained. Individuals from two other sites, CA-MNT-1482 (aka CA-MNT-1913) and CA-MNT-1489, came from Rancho San Carlos, adjacent to and south of the Carmel Valley. One burial was encountered at each site during monitoring of construction activities [11].

<u>Pebble Beach (California):</u> 750-300 BP

<u>CA-MNT-834</u>

- I11553: 750-300BP

Pebble Beach is an unincorporated coastal community on the Monterey Peninsula two km northwest of Carmel. CA-MNT-834 is described as a Late Period residential site on the shores of Carmel Bay. Investigations were conducted as part of a large data recovery project by Gary Breschini and Trudy Haversat in 2001-2002 prior to the site's destruction [12].

<u>Monterey (California):</u> 1055-925 calBP

<u>CA-MNT-1060</u>

- I11245: 1055-925 calBP (marine corrected): 897-1021 calCE (1075±15 BP, PSUAMS-4878)

The City of Monterey is located at the southern end of Monterey Bay, California. Skeletal remains were encountered in Unit 1, 20-30 cm, at CA-MNT-1060, which is located on the coast near the famous Cannery Row area. Although excavators Breschini and Haversat obtained dates for this site that indicated occupation at 4000-3600 BP, the tooth analyzed for this study indicates an interment between 1300-1200 BP, towards the end of the Middle Period [13].

<u>Pacific Grove (California):</u> 5495-1 BP

<u>CA-MNT-834</u>

- I8578: 5495-4900 calBP (marine corrected): 3476-3017 calBCE (Beta-215938)
- I11552: 285-1 calBP (marine corrected): 1666-1950 calCE (170±15 BP, PSUAMS-5885)

Pacific Grove is a coastal city at the north end of the Monterey Peninsula just west of the city of Monterey. CA-MNT-831 is the oldest documented site on the Monterey Peninsula. More than half of the 31 radiocarbon dates obtained from this site date to 5770-5550 BP. Prehistoric burials were encountered during utility trenching for a senior housing project, and led to an archaeological investigation by Gary

Breschini and Trudy Haversat [14]. Permission for ancient DNA study of these remains was obtained from Ella Rodriguez, the Most Likely Descendant designated by the Native American Heritage Commission.

<u>Castroville (California)</u>: 1300-1200 BP

<u>CA-MNT-1382</u>

- I11244: 988-1163 calCE (1065±, EZV-00225) (marine correction was not possible)

Castroville is an unincorporated community 13 km northwest of Salinas in northern Monterey County. CA-MNT-1382 covers an extensive terrace northwest of town. The site has been subjected to repeated and often large scale episodes of disturbance, including being intersected by State Highway 1. A substantial archaeological investigation was undertaken in 1991 for road widening. Site testing indicated that surviving archaeological deposits dated to the Middle and Late Periods [15]. Human skeletal remains were discovered by Breschini and Haversat during later construction disturbance at the site, and a human tooth was submitted for analysis with approval from the designated Most Likely Descendant.

<u>Calaveras County (California):</u> 1570-555 calBP

<u>CA-CAL-29</u>

- I15171: 650-550 BP
- I15172: 650-550 BP
- I15173: 655-555 calBP (marine corrected): 1296-1394 calCE (635±15 BP, PSUAMS-7678)
- I15174: 650-550 BP
- I15175: 650-550 BP
- I15176: 650-550 BP

<u>CA-CAL-13</u>

- I15170: 1570-1415 calBP (marine corrected): 381-535 calCE (1640±20 BP, PSUAMS-7615)

Individuals from Calaveras County came from disarticulated skeletal remains discovered in deposits in two deep limestone caves in the foothills of the central Sierra Nevada. Because of the broken nature of the remains, the prevailing interpretation is that the bones resulted from secondary deposition by being dropped into the caves from above [16]. The individuals analyzed from CA-CAL-29, Moaning Cave, were excavated by Phil Orr in 1951 [17]. These travertine-encrusted remains were from three male and three female individuals. A single genetically male individual from CA-CAL-13, Cave of the Skulls, also from came from Orr's investigations in 1951.

<u>Cerro De Trincheras, Sonora (Mexico):</u> 2855-500 calBP

- I23711: 960-795 calBP (marine corrected): 990-1155 calCE (990±25 BP, PSUAMS-12583)
- I23712: 750-500 BP
- I23710: 630-525 calBP (marine corrected): 1320-1425 calCE (550±20 BP, PSUAMS-12664)
- I23708: 750-500 BP
- I23709: 665-560 calBP (marine corrected): 1285-1390 calCE (655±20 BP, PSUAMS-12582)
- I23707: 650-550 calBP (marine corrected): 1300-1400 calCE (605±15 BP, PSUAMS-12553)
- I19505: 640-540 calBP (marine corrected): 1310-1410 calCE (580±20 BP, PSUAMS-12696)
- I8363: 750-500 BP
- I8242: 750-500 BP
- I8243: 750-500 BP

- I23706: 2773-2461 calBP (not marine corrected): 823-511 calBCE
- I23705: 2855-2760 calBP (marine corrected): 910-810 calBCE (2715±25 BP, PSUAMS-12672)
- I23704: 1935-1820 calBP (marine corrected): 15-130 calCE (1950±20 BP, PSUAMS-12863)

Cerro de Trincheras, Sonora
The Trincheras archaeological tradition (250-1450 CE) was the dominant cultural entity throughout the southern Sonoran Desert for the latter half of the pre-Columbian period. Mainly characterized by its distinctive purple-on-brown/red ceramics and the late type-site *cerros de trincheras* (or terraced hill), Cerro de Trincheras (1300-1450 CE) was the regional center of Late Prehispanic Period in the middle Magdalena Valley, with more than 900 terraces, ceremonial features, a plaza, and solstice observatories. During the El Cerro phase (1300-1450 CE), individuals were elaborately treated though cremation and placed in a wide variety of ceramic vessels and closely packed into a cemetery, often with several funerary objects. Although limited, inhumation burial may never have been fully replaced by cremation in the Trincheras tradition or was reserved only for some individuals. Physical evidence indicates that health declined considerably from Early Agriculture Period to the later portion of the sequence, particularly due to nutritional deficiencies. The transition to cremation rituals is a major shift in the cosmology of death, possibly shared with the Hohokam to the north, but is expressed uniquely at the Cerro de Trincheras in an urn cemetery and crematories.

La Playa, Sonora
The site of La Playa represents the primary Early Agricultural period site located within the Trincheras heartland. Adjacent to the Boquillas river, the site covers 10 km^2 and contains evidence for over 10,000 years of human occupation, from the terminal Pleistocene to the historic period. Work at the site over the past decades has documented the remains of hundreds of mortuary features that span much of the length of cultural development from the Late Archaic to the end of the prehistoric period. The Late Archaic/Early Agricultural period (2100 BCE – 50 CE) was a time when people adopted cultigens and developed horticultural economies with canal irrigation, resulting in substantial population growth, and increased social complexity in the region, including mortuary practices largely focused on inhumation burial. Individuals were often placed in flexed positions in houses, pre-existing pits, or small groups with limited funerary objects which were mostly reflective of personal decoration and a gendered identity.

Tayopa, Sahuaripa, Sonora (Mexico): 1750-450 BP
- I8235: 1450-550 BP
- I8236: 1450-550 BP
- I8237: 1450-550 BP
- I8238: 1450-550 BP
- I8239: 1450-550 BP
- I8240: 1450-550 BP
- I8241: 1450-550 BP
- I17108: 1450-550 BP
- I17096: 1450-550 BP
- I17109: 1450-550 BP
- I17114: 1450-550 BP
- I17125: 1450-550 BP
- I17113: 1450-550 BP
- I17112: 1450-550 BP

The Tayopa area is located in the Sierra Madre Mountain region of eastern Sonora. Like other parts of northwestern Mexico, Tayopa has received comparatively little archaeological investigation; researchers in the early 20th century through today have tended to put more time and resources into cultures found in the Valley of Mexico and Maya region. Therefore, much less is known about the people who inhabited the region prior to Spanish incursion than in other parts of Mexico.

One of the earliest intensive studies of northwest Mexico was the 1937-1940 Sonora-Sinaloa Archaeological Survey project led by Gordon F. Ekholm of the American Museum of Natural History (in New York), which produced collections that remain foundational to studies of the area. Unfortunately, outside of a few brief summary pieces of his research (e.g. Ekholm 1939 [18], 1940 [19]), Ekholm did not publish results of this project. As with other early researchers who worked in Mexican and Mesoamerican areas outside of the Valley of Mexico and Maya region, Ekholm was interested in understanding how northwest Mexico articulated with "classic," more well-known Mesoamerican cultures [20]. Thus, the main purpose of the Sonora-Sinaloa Archaeological Survey Main was to fill in the archaeological gap between American Southwest and northern Mesoamerican frontier [20]. To accomplish this, Ekholm collected material from the surface of 106 sites in Sonora (and also sites in Sinaloa), and excavated some larger sites. He also purchased existing collections in the region [21]. Ekholm had three field seasons (six months each) and was guided by local informants. He recorded sites in a variety of environmental settings, though many were in river valleys or near water sources [21]. Ekholm gave numbers to each of the sites he examined. Sites included in this project were number 59 (Sahuaripa), number 60 (Cueva de la momia), number 61 (Rancho Tayopa), number 62 (Cueva Tayopa), and number 63 (Paxon Hayes Mummy Burial).

This project uses individuals currently housed at the AMNH that were collected during Ekholm's Sonora-Sinaloa Archaeological Survey project. All individuals were sampled at the AMNH using the cranial base drilling method [22]. Of the individuals we studied, 14/16 produced working data (7/7 from site 59, 2/3 from site 60, 1/2 from site 62, and 4/4 from site 63). Contextual information for these sites and individuals is scant, though they likely date to ~1300-1600 CE, during the Postclassic period. The people who lived in Tayopa at this time were nomadic hunter-gatherers.

Morphological sex had been previously documented and recorded for six of the individuals included in this study. These assessments corresponded with genetic sex determinations in all cases but one (I17108 from site #60, who was classified as male morphologically but female genetically). Additionally, one individual (I17112) had coverage too low to make a genetic sex assessment.

Along with genetic sex, our analyses were able to determine that many of the Tayopa individuals were related, with some having relatives at different sites. I8239 (from site 59) and I17109 (from site 60) are father and daughter. I8239 (the father) is also a 2nd or 3rd degree relative of I8241 (from site 60), I17112 (from site 63), and I17108 (from site 60). I17108 is also a 1st degree relative of I17109 and of I17112. Additionally, I17125 (from site 63) and I18236 (from site 59) are 2nd or 3rd degree relatives.

<u>Cueva de los Muertos Chiquitos (Mexico):</u> 1850-950 calBP

- I11986: 1850-950 BP
- I11985: 1245-1065 calBP (marine corrected): 706-881 calCE (1225±20 BP, PSUAMS-5770)
- I11987: 1850-950 BP
- I11984: 1850-950 BP
- I11988: 1300-1175 calBP (marine corrected): 650-773 calCE (1340±20 BP, PSUAMS-5785)
- I12571: 1245-1070 calBP (marine corrected): 705-880 calCE (1220±15 BP, PSUAMS-12699)
- I12570: 1065-960 calBP (marine corrected): 885-990 calCE (1125±15 BP, PSUAMS-12698)

- I12572: 1180-970 calBP (marine corrected): 770-980 calCE (1150±20 BP, PSUAMS-12709)
- I12574: 1820-1630 calBP (marine corrected): 130-320 calCE (1825±20 BP, PSUAMS-12666)
- I11983: 1850-950 BP

La Cueva de Los Muertos Chiquitos is an archaeological cave site overlooking the Rio Zape region in Durango, Mexico. It dates to about 1350 BP and is referred to as the Loma San Gabriel culture area. It was partially excavated in the 1950s, revealing two burial assemblages underneath plastered adobe floors, one on top of the other comprising 25 infants and children and 3 adults [23]. These intentional and largely intact burials were placed on reed mats and wrapped in cloth shrouds and some were placed in ceramic ollas. Crandall and Thompson (2014) [24] argue that these two assemblages, comprised largely of infants and children between the ages of 6 months to 8 years old, were the result of the sacrifice of nonadults during periods of severe drought. Using ethnohistoric and archival data on Tephuan people living today in the area, this explanation for the simultaneous deaths of so many nonadults seems likely [25]. Most of the nonadults exhibited active cases of non-specific periosteal reactions on the cranium and long bones. At least 5 of the infants show signs of scurvy and possible rickets [26]. Archaeobotanical material, pottery, carved wood remnants, shell beads and pendants, turquoise and obsidian were found in the cave in addition to quids and coprolites [27,28]. Inhabitants of this region were "farmagers", using both foraging and hunting as well as farming as strategies for food procurement [28]. Loma San Gabriel communities were located in the center of a trade network bridging Central America to the US Southwest to the north, and to communities towards the east and west in northern Mexico [29].

<u>San Lorenzo and Coyote Cave, Coahuila (Mexico):</u> 1050-660 calBP

- I1867: 725-660 calBP (marine corrected): 1229-1287 calCE (750±20 BP, PSUAMS-1360)
- I1863: 2950-2550 BP
- I1864: 1050-910 calBP (marine corrected): 904-1038 calCE (1035±25 BP, PSUAMS-1359)

Two sites located in the state of Coahuila, Mexico—San Lorenzo Cave and Coyote Cave—were included in this study. Coahuila, in northeast Mexico, consists primarily of an arid desert landscape, crossed by the Sierra Madre mountains. Compared to other parts of Mexico and the US, Coahuila has received sparse archaeological investigation. Preservation at open air sites in Coahuila is poor; cave sites such as San Lorenzo and Coyote Cave have been key for archaeological interpretations.

Culturally, Coahuila lies northeast of the Mesoamerican frontier (considered to be sites such as La Cueva de Los Muertos Chiquitos and La Quemada) [30]. Archaeological survey and the few sites that have been excavated with stratigraphic control show a continuity through time in material culture. In other words, the native people who lived in Coahuila had broadly similar nomadic hunter-gatherer subsistence practices for the 10,000 or more year occupation of the region. Excavation of cave sites has revealed that the Indigenous people of Coahuila used caves for thousands of years, often as burial sites. Mummies from these caves are perhaps what Coahuila is most known for archaeologically. Cueva de Candelaria, excavated in the 1950s, is the best-known preserved cave in Coahuila; mummies from Cueva de Candelaria are prominently displayed in the Mexico's *Museo Nacional de Antropología*. Excavators encountered no fewer than 200 individuals in the cave and estimated that the actual number of individuals was closer to 1000. The majority of these individuals were deposited as bundles and were mummified [31]. The preservation of Cueva de la Candelaria was unique. In nearly all other cave sites cultural and chronological associations are uncertain due to disturbance of sites by looting and mining [30].

Coyote Cave and San Lorenzo Cave were first recorded and had materials collected from them in the 1880s, 70 years before Cueva de la Candelaria was excavated. Already by the 1880s, the two sites had been badly disturbed and looted, like most other cave sites in Coahuila. The individuals from San Lorenzo

and Coyote Caves were collected in the 1880s by Edward Palmer, an English botanist employed by the Peabody Museum of Archaeology and Ethnology [32]. The caves are part of the limestone mountain cave system found in western Coahuila. Coyote Cave, near the town of Torreón, was "discovered" in 1865 by a local native, who told Palmer that at that time there were many mummy burials in the cave laid side by side. However, between 1865 and 1880, when Palmer collected from the cave, "treasure hunters…brought out and broke up the bundles in search of gold" [32]. Additionally, the cave's roof "collapsed and filled up most of the main chamber" by 1880 (*ibid*). Thus, when Palmer visited the cave, he found only six mummy bundles and a few isolated bone fragments [32].

San Lorenzo Cave is north of Coyote Cave, near San Lorenzo de la Laguna. Like Coyote Cave, the burials had been badly disturbed when Palmer encountered them. In the 1860s the caves were mined for saltpetre, and miners destroyed and desecrated burials/mummies while working. Palmer found no intact burials, but scattered human remains consisting of fifteen skulls and a small number of various other skeletal fragments [32].

Though the burials of both caves had been looted and badly disturbed, the remaining bone was well preserved due to dry, stable climates inside the caves. According to Walter Taylor [30], after Palmer delivered the skeletal material from Coyote and San Lorenzo Caves to the Peabody Museum, the ancient individuals remained unstudied for years.

We sampled teeth of three individuals from Coyote Cave and one individual from San Lorenzo. All produced working dating except one of the individuals from Coyote Cave (Peabody #80-28-20/22823). Biomorphological analysis in 1880 of I1863 and I1864 (Coyote Cave) determined these individuals to be morphologically male, which is consistent with the genetic sex. Analysis classified I1867 from San Lorenzo as female, which also is consistent with the genetic sex.

References for Supplementary Information

1 Punzo, J. L. V., M.E. in *Discovering Paquimé* (ed P.E.; Whalen Minnis, M.E.) 57-65 (The University of Arizona Press and the Amerind Foundation, 2016).

2 McGuire, R. M. E. V. in *Enduring Borderlands Traditions: Trincheras Sites in Time, Space, and Society* (ed Suzanne K.; Fish Fish, Paul; Villalpando, M.E.) 167-174 (The University of Arizona Press, 2007).

3 Johnson, J. Chumash islanders during the protohistoric and historic periods. *Channel Islands National Park Archaeological Overvie w and Assessment. Department of the Interior, National Park Service, Channe l Islands National Park, Ventura, California*, 3-19 (2010).

4 Orr, P. C. *Prehistory of Santa Rosa Island*. (Santa Barbara Museum of Natural History, 1968).

5 Szromba, M. Confidential Report on Cultural Resources Services Provided for the Paradise Cove Property, Malibu, California. (Rincon Consultants, Ventura, 2022).

6 Orr, P. C. Review of Santa Barbara Channel Archaeology. *Southwestern Journal of Anthropology* **8**, 211-226 (1952).

7 Rogers, D. B. *Prehistoric man of the Santa Barbara coast*. Vol. 1 (Santa Barbara Museum of Natural History, 1929).

8 Lebow, C. *Patterns of Subsistence at CA-SBA-48*. (2020).

9 Erlandson, J. M., Rick, T. C. & Vellanoweth, R. L. *A Canyon Through Time: Archaeology, History, and Ecology of the Tecolote Canyon Area, Santa Barbara County, California*. (University of Utah Press, 2008).

10 Orr, P. C. *Archaeology of Mescalitan Island and customs of the Canalino*. (Santa Barbara Museum of Natural History, 1943).

11 Breschini, G. S. & Haversat, T. Ancient DNA—Modern Connections: Results of Mitochondrial DNA Analyses from Monterey County, California. *Pacific Coast Archaeological Society Quarterly40* **2**, 31-93 (2008).

12 Breschini, G. S. & Haversat, T. Radiocarbon dating and cultural models on the Monterey Peninsula, California. *Pacific Coast Archaeological Society Quarterly* **38** (2002).

13 Breschini, G. S. & Haversat, T. A revised culture sequence for the Monterey Peninsula Area, California. *Pacific Coast Archaeological Society Quarterly* **44**, 1-24 (2011).

14 Breschini, G. S. & Haversat, T. *An Offramp on the Kelp Highway: Archaeological Investigations at CA-MNT-831, Pacific Grove, Monterey County, California*. (Coyote Press, 2008).

15 Jones, T. L., Van Bueren, T., Grantham, S., Huddleson, J. & Fung, T. W. Archaeological Test Excavations for the State Highway 1 Widening Project near Castroville, Monterey County, California. *Caltrans Environ-mental Division. MS on file at the Northwest Information Center of the California Historical Resources Inven-tory, Sonoma State University, Rohnert Park* (1996).

16 Moratto, M. J. *California archaeology*. (Academic Press, 1984).

17 Orr, P. C. Excavations at Moaning Cave Department of Anthropology Bulletin 1. *Santa Barbara Museum of Natural History* (1952).

18 Ekholm, G. F. *Results of an Archeological Survey of Sonora and Northern Sinaloa*. (Sociedad Mexicana de Antropología, 1939).

19 Ekholm, G. F. The archaeology of northern and western Mexico. *The Maya and Their Neighbors*, 320-330 (1940).

20 Gallaga, E. 2004 Catalogue: archaeological material from the Gordon F. Ekholm (1937-40) archaeological project in Sonora, Mexico. (2004).

21 Gallaga, E. *A Landscape of Interactions During the Late Prehispanic Period in the Onavas Valley, Sonora, Mexico*. (Arizona State Museum The University of Arizona, 2013).

22 Sirak, K. A. *et al.* A minimally-invasive method for sampling human petrous bones from the cranial base for ancient DNA analysis. *Biotechniques* **62**, 283-289 (2017). https://doi.org:10.2144/000114558

23 Brooks, S. T. & Brooks, R. H. Paleoepidemiology as a possible interpretation of multiple child burials near Zape Chico, Durango, Mexico. *Across the Chichimec Sea: Papers in honor of J. Charles Kelley, JC Kelley, CL Riley, and BC Hedrick (eds.). Southern Illinois University Press, Carbondale, Illinois*, 96-101 (1978).

24 Crandall, J. J. & Thompson, J. L. Beyond victims: exploring the identity of sacrificed infants and children at La Cueva de Los Muertos Chiquitos, Durango Mexico (571–1168 AD). *Tracing Childhood: Bioarchaeological Investigations of Early Lives in Antiquity, University Press of Florida, Gainesville. University Press of Florida, Florida, USA*, 36-57 (2014).

25 Riley, C. L. & Winters, H. D. The prehistoric Tepehuan of northern Mexico. *Southwestern Journal of Anthropology* **19**, 177-185 (1963).

26 Crandall, J. J. Scurvy in the Greater American Southwest: Modeling micronutrition and biosocial processes in contexts of resource stress. *International Journal of Paleopathology* **5**, 46-54 (2014).

27 Jiménez, F. A. *et al.* Zoonotic and human parasites of inhabitants of cueva de los muertos chiquitos, Rio Zape Valley, Durango, Mexico. *Journal of Parasitology* **98**, 304-309 (2012).

28 Pucu, E., Russ, J. & Reinhard, K. Diet analysis reveals pre-historic meals among the Loma San Gabriel at La Cueva de Los Muertos Chiquitos, Rio Zape, Mexico (600–800 CE). *Archaeological and Anthropological Sciences* **12**, 1-14 (2020).

29 Foster, M. S. in *The Archaeology of West and Northwest Mesoamerica* 327-351 (Routledge, 2019).

30 Taylor, W. W. Archaic cultures adjacent to the northeastern frontiers of Mesoamerica. *Handbook of Middle American Indians* **4**, 59-94 (1966).

31 Krieger, A. D. Cueva de la Candelaria. Volume I. Luís Aveleyra Arroyo de Anda, Manuel Maldonado-Koerdell, and Pablo Martínez del Río, with the collaboration of Ignacio Bernal and Federico Elizondo Saucedo. Memorias del Instituto Nacional de Antropología e Historia, No. 5, Secretariá de Educación Pública, Mexico, 1956. 216 pp., 102 drawings and photographs (8 in color). $10.40 (130 pesos). *American Antiquity* **23**, 325-326 (1958).

32 Studley, C. A. *Notes upon human remains from the caves of Coahuila, Mexico*. Vol. 3 233-259 (Salem Press, 1884).
